# Improvement in binding and function of a monoclonal antibody against *Shigella flexneri* 3a O-antigen *via* phage display and whole-cell in-solution panning

**DOI:** 10.1016/j.jbc.2026.111405

**Published:** 2026-03-25

**Authors:** Nicholas L. Xerri, Sophia Pulido, Mateusz Kędzior, Paul Savarino, Torrey Williams, Robert M. Gallant, Robert W. Kaminski, Devin Sok, Hayden R. Schmidt

**Affiliations:** 1 IAVI Neutralizing Antibody Center, San Diego, California, USA; 2 Latham Biopharm Group, Cambridge, Massachusetts, USA; 3 Stripe Inc., San Francisco, California, USA; 4 Global Health Investment Corporation, New York, New York, USA

**Keywords:** affinity maturation, anti-polysaccharide mAb, gram-negative bacteria, phage display, *Shigella* flexneri

## Abstract

As rates of antimicrobial resistance (AMR) among bacterial pathogens continue to rise, the discovery and development of novel classes of therapeutics that can serve as alternatives or adjuncts to traditional small-molecule antibiotics, such as monoclonal antibodies (mAbs), is a public health priority. Some of the most promising antigen targets for antibacterial mAbs are surface polysaccharides such as O-antigen (O-Ag), a component of the lipopolysaccharide found on the outer membrane of gram-negative bacteria. However, developing mAbs against bacterial surface polysaccharides with sufficient breadth and potency to be clinically viable is difficult in part because antibodies against polysaccharides are generally low affinity, and the challenging biochemistry of polysaccharides often precludes further affinity maturation of mAbs against these targets *in vitro*. Here, we use a phage display library and a whole-cell in-solution panning strategy to successfully improve the affinity of a mAb against *Shigella flexneri* 3a O-Ag *in vitro* without requiring the purification of the target antigen. We demonstrate that a single mutation can improve apparent affinity as measured by ELISA by approximately 10-fold without detectably increasing polyreactivity, and increased apparent affinity correlates with enhanced potency in antibacterial effector function and anti-virulence assays. In addition, the most potent variants also gained increased breadth, successfully coordinating complement deposition and complement-independent opsonophagocytosis against *S. flexneri* 3b, a serotype weakly recognized by the parent mAb. Altogether, this work represents an important first step towards expanding the antibody engineering toolkit for bacterial surface polysaccharides, which will aid the development of novel mAb therapeutics against AMR bacterial pathogens.

Antimicrobial resistance (AMR) among pathogenic bacteria is a rapidly growing threat that necessitates the development of novel therapeutics to supplement existing antibiotics (1). Monoclonal antibodies (mAbs) may be complementary to antibiotics because they can prevent and treat AMR infections by both recruiting effector cells to kill bacteria *via* Fc-mediated effector functions (*i.e.*, complement-driven bactericidal activity or complement-independent opsonophagocytosis) and by directly interfering with bacterial virulence (2). Some of the most promising antigens for antibacterial immunotherapies are polysaccharides on the bacterial surface. Surface polysaccharides are well-validated targets for approved bacterial vaccines (*e.g.*, those against *Haemophilus influenzae* b, *Streptococcus pneumoniae*, *Neisseria meningitidis*, and *Salmonella typhi*) (3, 4, 5, 6, 7). This provides a strong rationale for developing anti-polysaccharide mAbs, and anti-polysaccharide mAbs against several bacterial species are effective in preclinical animal models (8, 9, 10, 11, 12, 13, 14, 15, 16, 17). However, to date, few of these mAbs against bacterial polysaccharides have been evaluated in clinical trials. Panobacumab, an IgM mAb against O-antigen (O-Ag) from serotype O11 *Pseudomonas aeruginosa* obtained promising results in a phase IIa trial among patients with pneumonia, though the effect size was modest and no control arm was present (18), so additional studies are required to fully assess clinical benefit. Gremubamab, a bispecific mAb targeting the exopolysaccharide PsI and the protein PcrV, has entered the clinic (19) and recently shown promise in treating *P*. *aeruginosa* infections in patients with bronchiectasis, though similarly, this was a proof-of-concept trial, and more studies are required to ensure reproducible clinical efficacy (20).

One reason for the small number of both clinical trials and anti-polysaccharide mAb candidates in the pipeline is that discovering and engineering anti-polysaccharide mAbs that have sufficient breadth and potency to be clinically viable is challenging. Breadth for a given anti-polysaccharide mAb is often too narrow for clinical use because the chemical structures of most surface polysaccharides are highly variable (even within the same bacterial species) (21). Bacterial strains of a given species are typically grouped into serotypes (often dozens or more) based on the specific chemical structure of key surface polysaccharides (such as the O-Ag, which is a component of gram-negative lipopolysaccharide), and antibody responses against these bacteria are usually serotype-specific (22, 23). In addition to breadth, potency is another challenge for anti-polysaccharide mAbs since B cells targeting repetitive polysaccharide antigens undergo T cell independent responses that generate short-lived plasma antibody responses, which are predominately IgM with minimal somatic mutation and low affinity for the target antigen (24). While there are examples of high affinity anti-bacterial polysaccharide mAbs that have undergone extensive affinity maturation isolated from the peripheral blood mononuclear cells of convalescent donors (16, 25), it is unclear how widespread this phenomenon is. In principle, additional high-potency mAbs against bacterial surface polysaccharides can be developed by engineering high-affinity variants of known anti-bacterial polysaccharide mAbs through *in vitro* affinity maturation. However, the chemical heterogeneity, inherent complexity of polysaccharide biochemistry, and the conformational flexibility of these polysaccharides (leading to a high entropic cost for binding for mAbs (26)) makes achieving *in vitro* affinity maturation of these mAbs difficult using standard approaches.

To date, relatively few successful attempts at *in vitro* affinity maturation of anti-glycan mAbs have been reported (27, 28, 29, 30, 31, 32, 33, 34, 35, 36). Most of these efforts have targeted glycans that are easier to purify and lack the variable, highly repetitive architecture of polysaccharides like O-Ag or capsular polysaccharides. To our knowledge, the only bacterial O-Ag or capsular polysaccharide for which successful *in vitro* affinity maturation has been reported is the O-Ag from *Salmonella* serogroup B. However, the impact that improving the affinity of these mAbs had on antibacterial effector functions was not explored (33, 34, 37). As a result, the relationship between antibody binding affinity and the potency of specific effector functions is less well-characterized for mAbs against bacterial polysaccharides than for those against mammalian glycans.

Here, we utilize a creative whole-cell in-solution phage display panning strategy, coupled with next-generation sequencing, to overcome these challenges and improve the affinity of a prototypical anti-O-Ag mAb specific for *Shigella flexneri* serotype 3a. We demonstrate that one to two mutations are sufficient to not only improve potency across effector functions against the target serotype, *S. flexneri* 3a, but also to expand breadth to serotype 3b. Importantly, a lack of polyreactivity against other bacterial strains or common polyreactivity reagents such as single-stranded DNA (ssDNA) indicates that these improvements in binding are specific. Interestingly, binding affinity and potency are generally correlated for most effector functions, but are not necessarily interdependent, particularly for complement-independent opsonophagocytosis. Altogether, these data provide a framework for affinity maturing mAbs against bacterial polysaccharides *in vitro* and demonstrate that (at least in this test case) few mutations are needed to significantly improve the breadth and potency of an anti-O-Ag antibody.

## Results

### hFlex3a2 functions as a chimeric huIgG1 but not as an scFv

**Figure 1:**
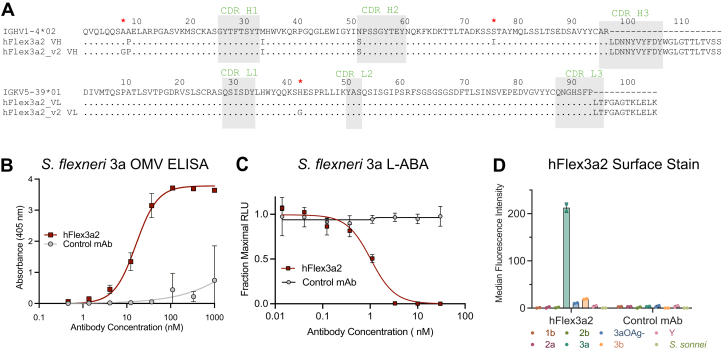
**Analysis of binding and function of putative hFlex3a2 as a huIgG1 chimera.***A*, alignment of putative hFlex3a2 VH and VL protein sequences to their closest germline progenitors shows high levels of similarity. Residues that were modified to create mAb hFlex3a2_v2 are denoted by (∗). Alignment and CDR predictions were determined with IMGT/V-Quest and rare residues were identified with abYsis (52, 53). *B*, titration ELISA measures the binding of increasing concentrations of hFlex3a2 huIgG1 to 2.5 μg/ml of *S. flexneri* 3a OMVs. This experiment is the average of three independent replicates each performed in duplicate. Error bars represent standard deviation. *C*, L-ABA measuring the ability of increasing concentrations of hFlex3a2 huIgG1 and a non-binding control mAb, to coordinate complement-mediated bacterial killing of *S. flexneri* 3a. L-ABA is the average of three biological replicates performed in duplicate. Error bars represent standard deviation. *D*, surface staining with 50 nM of mAb shows that hFlex3a2, and not a non-binding control mAb, binds specifically to *S. flexneri* 3a wildtype O-Ag. This experiment is the average of two experimental replicates. Error bars represent standard deviation.

hFlex3a2 is a murine hybridoma IgG3 that binds to *S. flexneri* 3a O-Ag and can coordinate complement-mediated bacteriolysis of this serotype in an antibody bactericidal assay (38). We determined the sequence of this mAb (Table S1) and found that it has many of the properties commonly associated with anti-O-Ag mAbs. The putative variable gene segment of the heavy chain of hFlex3a2 differs from the germline segment IGHV1-4∗02, its closest germline progenitor, by only four amino acids, while the putative variable gene segment of the light chain of hFlex3a2 is entirely identical to its closest germline segment IGKV5-39∗01 (Fig. 1*A*). The high degree of similarity to its closest predicted germline progenitor suggests that immunizing mice with purified lipopolysaccharide (LPS) did not stimulate high levels of somatic mutation *in vivo.* The putative VH and VL genes were grafted onto the human IgG1 constant regions to create a chimeric huIgG1, and this antibody was confirmed by ELISA to bind to *S. flexneri* 3a outer membrane vesicles (OMVs) (Fig. 1*B*), a proxy for the bacterial cell surface (39), as well as to coordinate complement-mediated bacteriolysis of *S. flexneri* 3a in a luminescent antibody bactericidal assay (L-ABA, Fig. 1*C*). Additionally, hFlex3a2 was highly serotype-specific, binding to only the surface of *S. flexneri* 3a that expresses wildtype O-Ag, when screened against a panel of serotypes and an O-Ag knockout mutant (Figs. S6*B* and 1*D*). Thus, the low level of somatic mutation, combined with its narrow breadth, makes hFlex3a2 representative of a canonical mAb against a bacterial surface polysaccharide.

**Figure 2:**
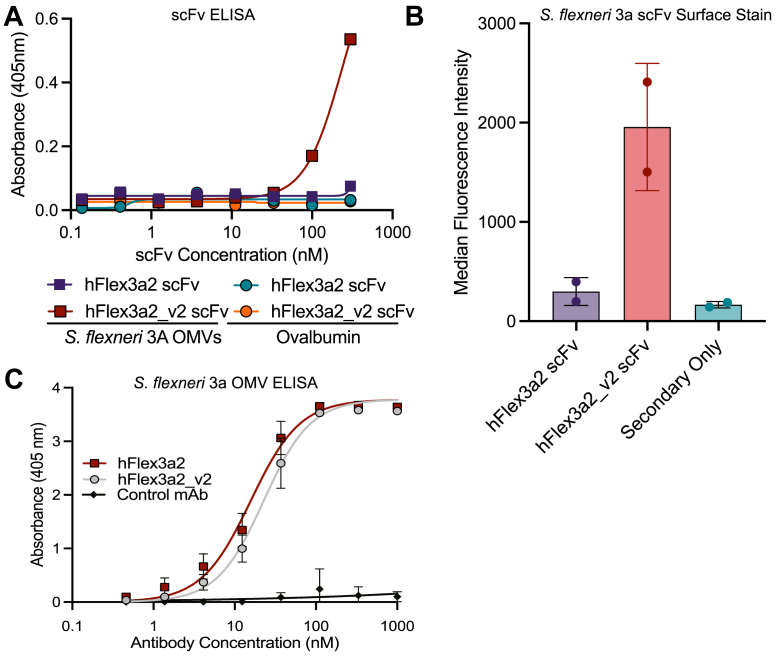
**Analysis of binding of hFlex3a2 and hFlex3a2_v2 as an scFv.***A*, titration ELISA shows that at concentrations above 100 nM of scFv, hFlex3a2_v2, but not hFlex3a2, binds to *S. flexneri* 3a OMVs (2.5 μg/ml). Neither scFv bound to the unrelated antigen ovalbumin (5 μg/ml). This experiment was repeated three times, and a representative experiment is shown. *B*, surface staining confirms that in the scFv format, hFlex3a2_v2, but not hFlex3a2, binds *S. flexneri* 3a. scFvs were tested at a concentration of 1 μM. This experiment represents two replicates. Error bars represent standard deviation. *C*, titration ELISA of hFlex3a2_v2 reformatted as a chimeric huIgG1 and the original hFlex3a2 huIgG1 shows similar binding to *S. flexneri* 3a OMVs (2.5 μg/ml). This experiment is the result of three independent replicates performed in duplicate. Error bars represent standard deviation.

We sought to improve the affinity of hFlex3a2 against *S. flexneri* 3a O-Ag using phage display. Since hFlex3a2 would be displayed as an scFv, we wanted to confirm that the antibody retains functionality in this format. However, binding of *S. flexneri* 3a OMVs by hFlex3a2 in an scFv format could not be detected by ELISA (Fig. 2*A*). This is not surprising, as scFvs often exhibit lower target affinity and are less stable than their IgG counterparts (40, 41). To restore function to hFlex3a2 as an scFv, we identified two residues in the framework region of the VH and one in the VL that were rare and thus may be potentially problematic (42). We mutated all three of these residues to the amino acid most frequently found at their respective positions when compared to a database of other murine antibodies. We called this new mAb hFlex3a2_v2 (Fig. 1*A*). Two of these new mutations were away from the closest germline sequence, while the other reverted that position back to its predicted germline amino acid (Fig. 1*A*). As an scFv, binding of hFlex3a2_v2 to *S. flexneri* 3a OMVs, but not to the unrelated antigen ovalbumin, was observed (Fig. 2*A*). Antigen binding was further confirmed by surface staining, which demonstrated that hFlex3a2_v2 scFv, but not the original hFlex3a2 scFv binds to the surface of *S. flexneri* 3a (Fig. 2*B*). Importantly, as a chimeric huIgG1, hFlex3a2_v2 exhibited antigen binding similar to the parent mAb, demonstrating that the rare residues we identified did not impact IgG antibody binding (Fig. 2*C*). Since hFlex3a2_v2 retained binding as a huIgG1 and restored binding as an scFv while retaining antigen specificity, affinity maturation by phage display was completed using the hFlex3a2_v2 background.

### Whole-cell in-solution panning enriched for mutations in the VL

**Figure 3:**
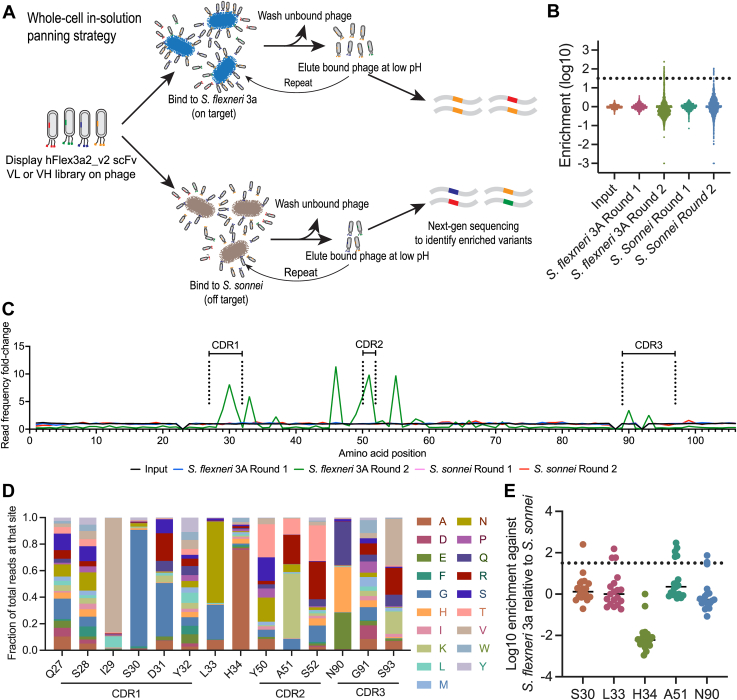
**Whole-cell in-solution panning enriches for VL variants.** A, Whole-cell in-solution panning strategy. To identify better binding variants of hFlex3a2_v2 scFv, we panned a phage-displayed site-saturated mutagenesis scanning library of VL or VH against either *S. flexneri* 3a or *S. sonnei* in-solution for two rounds and sequenced output phage by next-generation sequencing to identify variants that were specifically enriched against *S. flexneri* 3a. *B*, log10 enrichment of variants in the VL library after two rounds of panning against *S. flexneri* 3a and *S. sonnei*. The dotted line is 20 standard deviations above the mean mutation enrichment in the input library and indicates a stringent threshold set to distinguish bacteria-mediated enrichment from background variability. *C*, fold-change in the frequency of next-generation sequencing reads for variant mutations at each site on hFlex3a2_v2 VL after panning, relative to their frequency in the initial library, shows that certain residues that cluster in and around the CDRs have a large increase in read frequency. *D*, The percentage of distribution of all variants for each site of the CDRs after two rounds of panning against *S. flexneri* 3a shows that at some sites (I29, S30, H34) a single variant, or at other sites (L33, A51, N90, S93) a handful of variants were enriched for. *E*, specificity of mutational enrichment was determined. Each dot represents the enrichment of a variant against *S. flexneri* 3a relative to *S. sonnei* at the indicated residue after two rounds of panning. Positive numbers indicate variants that were enriched against *S. flexneri* 3a relative to *S. sonnei*, zero indicates similar enrichment against both bacterial species, and negative numbers indicate variants that were enriched for against *S. sonnei* relative to *S. flexneri* 3a. Dotted line is 20 standard deviations above the mean mutation enrichment in the input library.

To test whether phage display can be used to identify single mutations that improve hFlex3a2_v2 affinity, we designed two double-barcoded site-saturated mutagenesis scanning libraries: one for VH and one for VL (43). Each library contained single amino acid substitutions of every amino acid besides cysteine at every site of either the VH or VL, leading to total library sizes of 2223 and 1995 variants for the VH and VL libraries, respectively. Flanking synonymous codons were used to barcode mutation sites, enabling bioinformatic identification of sampled positions for mutation enrichment analysis. Initial efforts to fix purified *S. flexneri* 3a lipopolysaccharide to a plate for selection *via* solid-phase panning were unsuccessful. Whole-cell in-solution panning was previously used to discover antibodies that bind to bacterial surface carbohydrates (44), and so we adopted a similar panning strategy for affinity maturing our existing O-Ag antibody (Fig. 3*A*). The phage display libraries were panned in solution for two rounds against either the target bacterium *S. flexneri* 3a or *Shigella sonnei*, which is a closely related bacterium with an entirely different O-Ag structure (45). Input and output page libraries were subsequently sequenced by next-generation sequencing. We reasoned that variants enriched specifically after panning against *S. flexneri* 3a, but not *S. sonnei*, were likely to exhibit improved binding to the target relative to the parental mAb, and these variants could be evaluated further. This approach also had some advantages over other panning strategies. Specifically, the approach did not require large-scale purification of O-Ag and also allowed for panning on O-Ag in its native conformation and abundance without any biochemical modifications.

After two rounds of panning with the VH library, no enrichment of hFlex3a2_v2 variants was detected (Fig. S1*A* and S1*B*). In contrast, specific enrichment did occur for variants in the VL library. Despite all the variants being of roughly equal abundance in the input library, by the second round of panning, positively and negatively enriched variants were identified when panned against *S. flexneri* 3a as well as *S. sonnei* (Fig. 3*B*). After two rounds of panning against *S. flexneri* 3a, a strong increase in the frequency of next-generation sequencing reads for variant mutations mapping to residues that cluster around the predicted VL CDRs was observed (Fig. 3*C*). This enrichment was driven by the accumulation of reads mapping to either a single variant or a subset of variants at these sites (Fig. S2). In some cases, such as at residue S30, a single variant was found to dominate relative to other variants with mutations at that site, while at other residues, such as N90, a few variants were found to be similarly abundant (Fig. 3*D*). To determine whether highly enriched variants identified during phage display screening were of higher affinity than the parental antibody, we picked four sites—S30, L33, A51, and N90— or further investigation. We chose these sites because all four had large increases in read frequencies after panning, suggesting that these residues may be important. Additionally, each variant was specifically enriched following panning against *S. flexneri* 3a relative to *S. sonnei* (Figs. 3*E*, and S3, and Table 1). Lastly, these variants are located in or adjacent to CDRL1 (S30 and L33), CDRL2 (A51) or CDRL3 (N90). Since no variants in the VH were enriched for, we will refer to the hFlex3a2_v2 variants solely by their VL mutation(s) from here on.

**Table 1:** Summary of functional metrics for hFlex3a2 variants

Antibody	3a fold enrichment	Sonnei fold enrichment	3a ELISA EC50 nM (95% confidence interval)	3a L-ABA IC50 (nM)	3a OPA (%CTV+)	3a Inv. Assay (% of PBS)	3b ELISA EC50 nM (95% confidence interval)	3a L-ABA IC50 (nM)	3b OPA (%CTV+)	3b Inv. Assay (% of PBS)
hFlex3a2_v2	—	—	6.9 (6.4–7.5)	1.9	17.7	118.3	325 (266.5–434.3)	26.3	7.4	151.7
S3G	243.5	1	1.5 (1.4–1.6)	1.4 (*p* = 0.01)	25.6	34.5 (*p* < 0.001)	24.7 (22.9–26.6)	3.8 (*p* < 0.001)	12.6	—
L33N	129.9	0.8	0.8 (0.7–0.8)	1.3 (*p* = 0.04)	33.7 (*p* = 0.01)	19.8 (*p* < 0.001)	74.8 (68.3–82.5)	1.6 (*p* < 0.001)	22.8 (*p* = 0.002)	121.3
H34A	15.9	61.2	38.4 (36.1–41)	4.9 (*p* < 0.001)	21.8	—	—	141.7 (*p* = 0.003)	11	—
A51R	128.5	0.7	1.8 (1.7–1.9)	1.4 (*p* = 0.04)	29.6	44.4 (*p* < 0.001)	207.5 (188–232.7)	24	28.7 (*p* < 0.001)	—
N90H	105.2	3.7	5.7 (5.4–6)	1.6	32.2 (*p* = 0.03)	—	899.4 (527.3–3091)	91.7 (*p* = 0.005)	26.9 (*p* < 0.001)	—
S30G + L33N	—	—	0.7 (0.66–0.74)	0.7 (*p* < 0.001)	38.6 (*p* < 0.001)	32.8 (*p* < 0.001)	61.3 (58.2–64.8)	6.2 (*p* < 0.001)	31.3 (*p* < 0.001)	93.33
S30G + A51R	—	—	1 (0.9–1)	0.6 (*p* < 0.001)	29.7	15.7 (*p* < 0.001)	57 (53–61.5)	7.8 (*p* < 0.001)	33.4 (*p* < 0.001)	—
L33N + A51R	—	—	2.4 (2.3–2.6)	0.9 (*p* < 0.001)	27.6	30.1 (*p* < 0.001)	90.6 (81.8–101.3)	11.5 (*p* = 0.03)	25.3 (*p* < 0.001)	—
S30G + L33N + A51R	—	—	1 (1–1.1)	0.8 (*p* < 0.001)	25.4	18.7 (*p* < 0.001)	54.9 (51.2–59.1)	6.5 (*p* < 0.001)	25.4 (*p* < 0.001)	—
All 5	—	—	71.6 (65–79.2)	4.5 (*p* < 0.001)	18.5	—	—	—	4.1	—

A summary of key functional metrics for hFlex3a2 and variants in binding and functional assays. These data include the relative enrichment in the scFv library following two rounds of panning against either *S. flexneri* 3a or *S. sonnei*, EC50 values in ELISA binding to OMVs from *S. flexneri* 3a or 3b, L-ABA IC50 values (nM), OPA uptake (%CTV + macrophages with 30 nM mAb), and the invasion results against *S. flexneri* 3a or 3b as a percentage of PBS invasion. *P*. values < 0.05, indicating a significant difference in comparison to the parent mAb hFlex3a2_v2 using one-way repeated measure ANOVA with Dunnett’s test for multiple comparisons are shown.

### Variants enriched in phage display improve mAb binding to *S. flexneri* 3a O-Ag as chimeric huIgG1s

We hypothesized that the selected variants of hFlex3a2_v2 that were highly enriched against *S. flexneri* 3a but not *S. sonnei* (S30G, L33N, A51R, and N90H) were likely to bind *S. flexneri* 3a O-Ag better than the parent mAb. For a comparison, we chose one additional variant — H34A — that was highly enriched against both *S. flexneri* 3a and *S. sonnei* (Figs. 3, *D* and *E*, and S3, and Table 1). All the selected variants were produced as chimeric huIgG1s for further characterization (Table 1).

**Figure 4:**
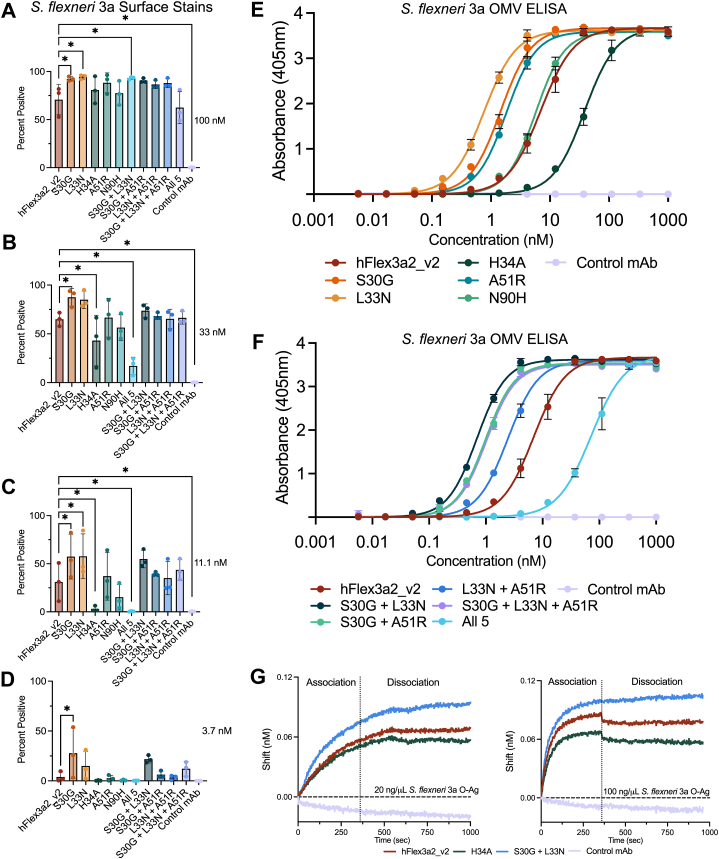
**Binding analysis of hFlex3a2_v2 variants specifically enriched against *S. flexneri* 3a and produced as huIgG1 chimeras surface.** Staining experiment showing the percent of *S. flexneri* 3a bound by hFlex3a2_v2 variants at mAb concentrations of (*A*) 100 nM, (*B*) 33 nM, (*C*) 11.1 nM, and (*D*) 3.7 nM. Surface staining is the average of three biological replicates. Error bars represent standard deviation. One-way repeated measure ANOVA with Dunnet’s test for multiple comparisons was performed. *p* values can be found in Table 1 and those < 0.5 are denoted by a ∗. *E*, binding curves from a titration ELISA against *S. flexneri* 3a OMVs (2.5 μg/ml) show relative binding potencies of hFlex3a2_v2 single amino acid variants or (*F*) hFlex3a2_v2 combinatorial amino acid variants. ELISA binding curves are the average of three independent replicates each performed in duplicate. ELISAs in panels (*E*) and (*F*) were performed in parallel but graphed separately for clarity. hFlex3a2_v2 and Control mAb data is the same in these two panels. *G*, binding of hFlex3a2_v2, H34A, S30G + L33N, and a non-binding control as huIgG1s to purified *S. flexneri* 3a O-Ag was assessed by BLI. This experiment is the average of two experiments performed with independently purified batches of mAbs.

To assess the relative binding strength of the hFlex3a2_v2 variants, we performed surface staining and quantified the percentage of *S. flexneri* 3a that was positive for mAb binding at each of concentrations of 100 nM, 33 nM, 11.1 nM, and 3.7 nM by flow cytometry (Fig. S4, *A* and *B*). At higher concentrations of mAb, all the variants and the parent mAb bound to *S. flexneri* 3a. Staining with variants S30G and L33N showed a subtle increase in the percentage of cells bound relative to the percentage of those bound with the parent mAb, while H34A bound a decreased percentage of cells (Figs. 4, *A* and *B*, and S4*B*). This trend remained true at lower concentrations (Figs. 4, *C* and *D*, and S4*B*). Together, this suggests that some of the variants identified in phage display may bind *S. flexneri* 3a O-Ag better than the parent mAb. No staining was detected against *S. flexneri* serotype 2a, confirming the serotype specificity of hFlex3a2_v2 and its variants in this assay (Fig. S7*B*).

To further investigate binding, we performed a titration ELISA, which confirmed that all of the hFlex3a2_v2 variants bind to *S. flexneri* 3a OMVs (Fig. 4*E*) and showed that three of the variants that were specifically enriched against *S. flexneri* 3a (S30G, L33N, and A51R) had higher apparent affinities than the parental mAb hFlex3a2_v2, while the variant that was enriched against both *S. flexneri* 3a and *S. sonnei* (H34A) had a lower apparent affinity. The ELISA binding curves were used to calculate EC_50s_ (Table 1 and Fig. 4*E*). The parent mAb hFlex3a2_v2 had an EC_50_ of 6.9 nM. L33N, the variant with the highest apparent affinity as measured by ELISA, had an EC_50_ of 0.8 nM, while variant H34A had the lowest apparent affinity (EC_50_ 38.4 nM). Binding improvements were specific for the target antigen, as no binding by the hFlex3a2_v2 variants were detected in an ELISA performed in parallel against *S. flexneri* antigen IpaD (Fig. S5*A*). Overall, the *S. flexneri* 3a OMV ELISA corroborated whole-cell surface staining results (Fig. 4, *A*–*D*) and is consistent with the hypothesis underlying our panning strategy, that specific enrichment against *S. flexneri* 3a provides a means of identifying variants that improve target affinity.

We next wanted to determine whether combining the most beneficial single amino acid mutations—S30G, L33N, and A51R—would synergize with one another to further improve the affinity of hFlex3a2_v2. We constructed all the possible double and triple combinatorial mutants. Since all the combinatorial variants retained their ability to bind *S. flexneri* 3a (Fig. 4, *A*–*D*), we performed an ELISA to determine their apparent affinities in this assay. Two of the combinatorial variants, S30G + L33N and S30G + A51R, had synergistic improvements in ELISA apparent affinity, meaning that the combinatorial variant had a lower EC_50_ than that of either single mutation contained in the combination (Table 1, and Fig. 4*F*). Variant S30G + L33N was the most potent binder, with an EC_50_ = 0.7 nM. We also tested a variant combining all five of the single mutations and found that its apparent affinity (EC_50_ = 71.6 nM) was lower than the parent mAb, similarly to H34A alone, suggesting that the presence of mutation H34A may be dominantly detrimental over the other beneficial mutations. Due to the oligomeric structure of O-Ag, it is difficult to accurately measure its molar concentration in solution, complicating efforts to precisely measure the binding kinetics between the mAbs and purified O-Ag *in vitro*. However, biolayer interferometry (BLI) confirmed that the parent mAb, as well as variants S30G + L33N and H34A, can all bind purified *S. flexneri* 3a O-Ag (Fig. 4*G*). Consistent with the selected mutations affecting binding behavior, at both concentrations of O-Ag tested, S30G + L33N reached a higher plateau in BLI than the parental mAb, while H34A reached a lower plateau. Lastly, a panel of polyspecificity reagents were used in ELISA to demonstrate that the mAb variants had limited polyreactivity against the antigens tested, confirming that the better binding variants retained their antigen specificity (Fig. S5*B*).

### Improving binding of hFlex3a2_v2 enhances effector and anti-virulence functions

**Figure 5:**
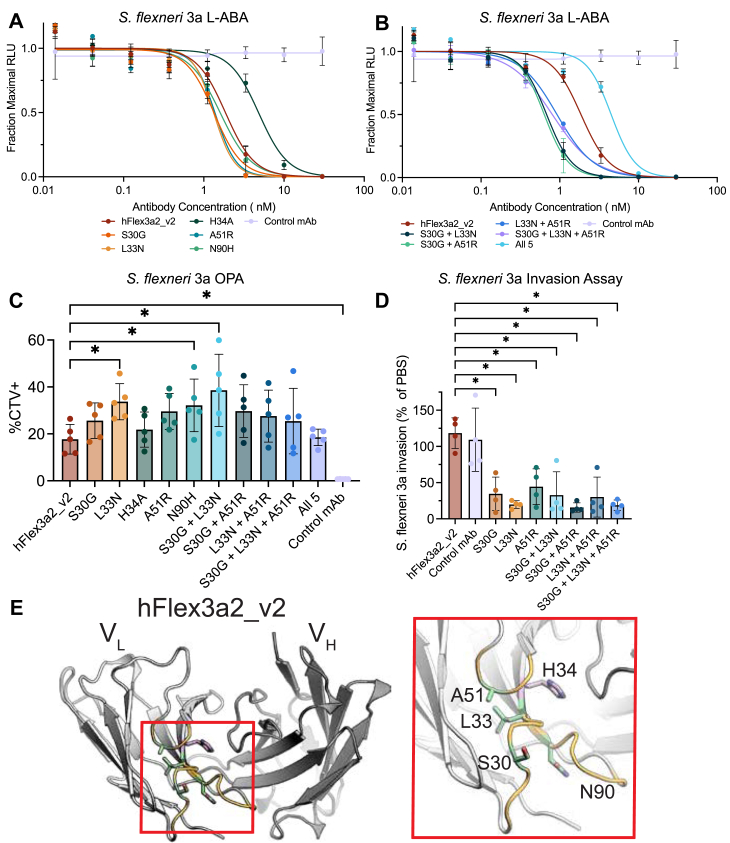
**Functional analysis of hFlex3a2_v2 variants against *S. flexneri* 3a.***A*, L-ABA assay comparing the ability of single hFlex3a2_v2 variants or (*B*) combinatorial hFlex3a2_v2 variants and a non-binding control to coordinate complement-mediated bacteriolysis of *S. flexneri* 3a. L-ABA is the average of three biological replicates each performed in duplicate. L-ABAs in panels (*A*) and (*B*) were performed in parallel but graphed separately for clarity. hFlex3a2_v2 and control mAb data is the same in these two panels. *p* values in Table 1 were calculated on logIC_50_ values by repeated measure Anova with Dunnett’s correction for multiple comparisons. *C*, OPA assay measures the percentage of THP-1 cells that have phagocytosed *S. flexneri* 3a that was opsonized with 30 nM of the indicated mAb. This experiment is the result of five biological replicates each performed in duplicate, and *p* values were calculated by repeated measure ANOVA with Dunnett’s correction for multiple comparisons. Error bars represent standard deviation. *D*, Invasion assay shows the percent invasion of *S. flexneri* 3a incubated with 1 μM of the indicated mAb normalized to the invasion rate of *S. flexneri* 3a in PBS. This experiment is the result of four biological replicates each performed in duplicate, and *p*-values were calculated by repeated measure ANOVA with Dunnett’s correction for multiple comparisons. Error bars represent standard deviation. For all data, *p* values can be found in Table 1 and those < 0.5 are denoted by a ∗. (*E*) A cartoon depicting the top-ranked Alphafold model of hFlex3a2_v2 (pLDDT = 96. 2, pTM = 0.929, and ipTM = 0.909). The VL is shown in light gray, and the VH is shown in dark gray. CDRL loops are colored orange, and residues where variants were selectively enriched against *S. flexneri* 3a following panning against *S. flexneri* 3a and *S. sonnei* and were experimentally characterized are colored *green*, while H34 (not selectively enriched) is shown in *pink*.

**Figure 6:**
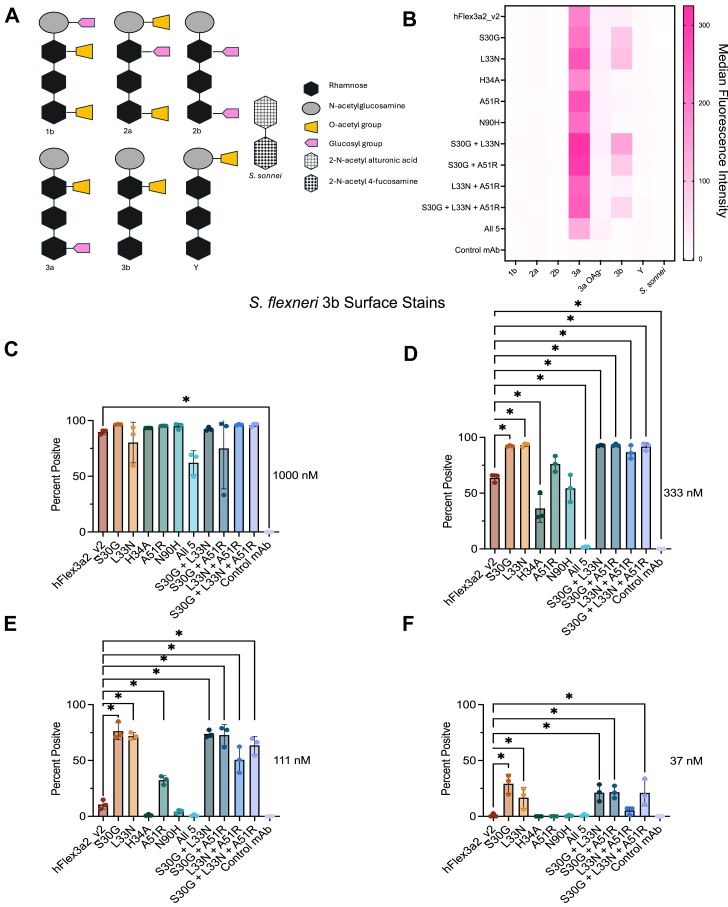
**Binding analysis of hFlex3a2_v2 variants against *S. flexneri* 3b.***A*, schematic depicting the O-Ag structures of our panel of *S. flexneri* serotypes. *B*, surface staining with 50 nM of hFlex3a2_v2 variants and a non-binding control mAb to a panel of *S. flexneri* and *S. sonnei* serotypes. This experiment is the average of two replicates. Surface staining experiment showing the percentage of *S. flexneri* 3b bound by hFlex3a2_v2 variants at mAb concentrations of (*C*) 1000 nM, (*D*) 330 nM, (*E*) 111 nM and (*F*) 37 nM. Surface staining is the average of three biological replicates. Error bars represent standard deviation. One-way repeated measure ANOVA with Dunnet’s test for multiple comparisons was performed. *p* values can be found in Table 1, and those < 0.5 are denoted by a ∗.

To probe the relationship between improved O-Ag affinity and potency across antibacterial functions, we tested our panel of hFlex3a2_v2 variants in a series of effector function and virulence assays. A Luminescent Antibody Bactericidal Assay (L-ABA), which measures the ability of antibodies to coordinate complement-mediated bacteriolysis, showed that the parent antibody was already potent at this effector function (IC_50_ = 1.9 nM Fig. 5*A* and Table 1). Nonetheless, the best binding single amino acid variant, L33N, modestly but reproducibly improved upon the parent mAb (IC_50_ = 1.3 nM, *p* = 0.04), while the best binding combinatorial variant, S30G + L33N, had a more substantial ∼2.7-fold improvement in potency (IC_50_ = 0.7 nM, *p* < 0.001, Fig. 5*B* and Table 1). The worst binding variant H34A had the weakest potency in L-ABA (IC_50_ = 4.9 nM, *p* < 0.001, Fig. 5*A*). Similarly, when hFlex3a2_v2 was tested in a complement-independent opsonophagocytosis assay (OPA) at a 30 nM concentration, 17.7% of monocytes were cell trace violet positive (CTV+), indicating that they had phagocytosed *S. flexneri* 3a that was opsonized with this mAb (Fig. 5*C* and Table 1). The gating strategy for OPA is shown in Figure 6*A*. Although hFlex3a2_v2 already had the ability to facilitate OPA activity, L33N, the best binding single amino acid variant, was almost 2-fold more potent at the same concentration (33.7% CTV+, *p* = 0.01). The best binding combinatorial variant S30G + L33N was the most potent at facilitating OPA (38.6% CTV+, *p* < 0.001). Despite having little improvement in binding and L-ABA compared to the parental mAb, variant N90H was the third most potent in OPA (32.2% CTV+, *p* = 0.03). Similarly, despite exhibiting lower apparent affinity, H34A and the combination of all five mutations had similar potency in OPA to the parent antibody at 30 nM (Fig. 5*C*). This demonstrates that, while largely correlated, other factors beyond binding strength also impact O-Ag mAb function in the OPA.

In addition to promoting effector functions that modulate the immune system, mAbs can also directly neutralize bacterial virulence. As an intracellular pathogen, invasion of colonic epithelial cells is an indispensable step in the pathogenesis of *S. flexneri*. An *in vitro* invasion assay showed that, relative to PBS or a non-binding control mAb, hFlex3a2_v2 does not significantly block *S. flexneri* 3a invasion of a monolayer of HeLa cells (Fig. 5*D*). However, the highest affinity single amino acid variants and combinatorial variants all blocked *S. flexneri* 3a invasion by at least 60% (Fig. 5*D*). This demonstrates that for anti-O-Ag antibodies, increasing affinity can help to directly neutralize bacterial virulence.

To better understand the mechanistic basis for these improvements in binding and function, we generated a model of the hFlex3a2_v2 VH/VL complex using Alphafold two through ColabFold (Fig. S8) (46). Unsurprisingly, the predicted structure shows that all four specifically enriched sites tested here (S30, L33, A51, and N90) are all located near one another in three-dimensional space (Fig. 5*E*). S30, A51, and N90 are all solvent-accessible residues on the side chain, suggesting that S30 G, A51 R, and N90H may offer improved binding or functional potency by changing the interaction interface between hFlex3a2 and O-Ag. However, we cannot rule out indirect effects of these mutations on overall VL/CDR conformation that may simply make the mAb-ligand interaction more energetically favorable without direct interaction between these sites and O-Ag. In contrast, L33 is predicted to be buried in the VL, suggesting that this amino acid is unlikely to interact with O-Ag and that the improvements in binding and function driven by L33N most likely come from stabilizing the O-Ag-binding conformation of the hFlex3a2 VL, rather than directly interacting with O-Ag. Similarly, H34 is located at the VH/VL interface, suggesting that the deleterious effects on binding and function observed for H34A are most likely derived from alterations to overall mAb conformation or stability. This mutation may have been favorable for phage display of the hFlex3a2_v2 scFv (explaining why it was enriched regardless of target antigen) but may alter the conformation of the mAb format in a way that is detrimental to binding.

### Improving the affinity of hFlex3a2_v2 against *S. flexneri* 3a increases its functional potency against *S. flexneri* 3b

Lastly, we wanted to determine whether higher-affinity variants of hFlex3a2_v2 would also have increased breadth compared to their parent mAb. Although the precise epitope of hFlex3a2_v2 is not known, we reasoned that many of the components of *S. flexneri* 3a O-Ag are also found in the O-Ag of other *S. flexneri* serotypes, and some of the variants may have increased affinity against some O-Ag component common to other *S. flexneri* serotypes. Since achieving relevant breadth is often the greatest clinical limitation for anti-O-Ag mAbs, an increase in functional breadth is a potentially more important outcome of affinity maturation campaigns than increased potency against a single serotype.

We performed a surface staining experiment with a panel of *S. flexneri* serotypes that all share the same core O-Ag sugars, but with different modifications, as well as *S. sonnei*, which has an entirely unrelated O-Ag composition (Fig. 6*A*). As previously shown, all hFlex3a2_v2 variants bind to *S. flexneri* 3a and binding to a mutant *S. flexneri* 3a O-Ag- strain (Fig. S6*B*) was 10-fold lower than to *S. flexneri* 3a that expresses wildtype O-Ag, confirming that O-Ag is necessary for binding. For all the hFlex3a2_v2 variants tested, no reactivity to *S. flexneri* serotypes 1b, 2a, 2b, Y, or *S. sonnei* was observed (Fig. 6*B*). In contrast, surface staining against *S. flexneri* 3b showed that hFlex3a2_v2 weakly cross-reacts with this serotype. To better characterize the relative binding strength of different hFlex3a2_v2 variants, we performed surface staining against *S. flexneri* 3b using several dilutions of mAb (Figs. 6, *C*–*F*, and S7*A*). At 1000 nM concentration of mAb, a similar percentage of *S. flexneri* 3b was bound by the antibody, regardless of the variant tested, confirming weak cross-reactivity to this strain (Fig. 6*C*). However, at lower concentrations of mAb, a higher percentage of *S. flexneri* 3b were bound by antibodies when incubated with the highest affinity single and combinatorial variants, suggesting that these variants bind *S. flexneri* 3b better than the parent antibody (Figs. 6, *D*–*F*, and S7*A*).

**Figure 7:**
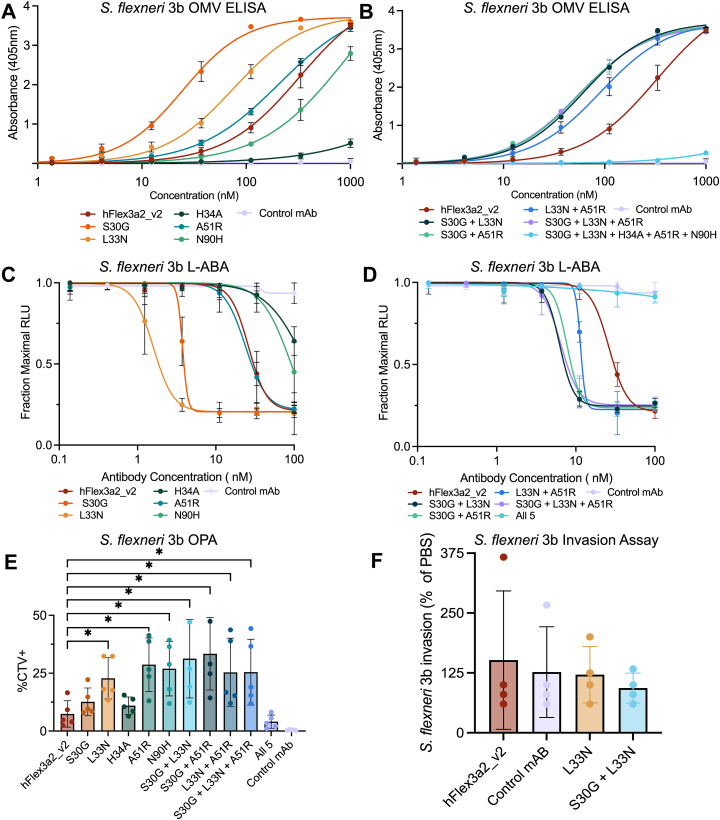
**Functional analysis of hFlex3a2_v2 variants against *S. flexneri* 3b.** Binding curves from a titration ELISA against *S. flexneri* 3b OMVs (2.5 μg/ml) show relative binding potencies of hFlex3a2_v2 (*A*) single amino acid variants or (*B*) hFlex3a2_v2 combinatorial amino acid variants. ELISA binding curves are the average of three independent replicates each performed in duplicate. ELISAs in panels (*A*) and (*B*) were performed in parallel but graphed separately for clarity. hFlex3a2_v2 and control mAb data is the same in these two panels. (*C*) L-ABA assay comparing the ability of single hFlex3a2_v2 variants or (*D*) combinatorial hFlex3a2_v2 variants and a non-binding control to coordinate complement-mediated bacteriolysis of *S. flexneri* 3b. L-ABA is the average of three biological replicates each performed in duplicate. L-ABAs in panels (*C*) and (*D*) were performed in parallel but graphed separately for clarity. hFlex3a2_v2 and control mAb data is the same in these two panels. *p*-values were calculated on logIC_50_ values by repeated measure Anova with Dunnett’s correction for multiple comparisons (*E*) OPA assay measures the percent of THP-1 cells that have phagocytosed *S. flexneri* 3b that was opsonized with 30 nM of the indicated mAb. This experiment is the result of five biological replicates each performed in duplicate, and *p*-values were calculated by repeated measure ANOVA with Dunnett’s correction for multiple comparisons. Error bars represent standard deviation. *F*, invasion assay shows the percent invasion of *S. flexneri* 3b incubated with 1 μM of the indicated mAb normalized to the invasion rate of *S. flexneri* 3b in PBS. This experiment is the result of four biological replicates each performed in duplicate. Error bars represent standard deviation. For all plots, *p* values can be found in Table 1 and those < 0.5 are denoted by a ∗.

Since surface staining experiments showed that some variants of hFlex3a2_v2 likely have improved binding potency against *S. flexneri* 3b, we wanted to further characterize their binding and function against this serotype. ELISA measuring binding to *S. flexneri* 3b OMVs, in agreement with the surface staining experiment, showed that hFlex3a2 weakly binds to *S. flexneri* 3b OMVs (Fig. 7*A*). Additionally, the variants showed similar results against *S. flexneri* 3b as against *S. flexneri* 3a. Specifically, single amino acid variants S30G and L33N increased apparent affinity, while H34A decreased apparent affinity as measured by ELISA (Fig. 7*A*, Table 1). All combinatorial variants improved binding relative to the parent mAb, with the exception of the combination of all five mutations, which, similarly to H34A alone, bound poorly to *S. flexneri* 3b OMVs (Fig. 7*B*).

We next wanted to determine whether these improvements in affinity for *S. flexneri* 3b O-Ag are sufficient to drive effector functions against this serotype. We compared all the hFlex3a2_v2 variants in an L-ABA assay. In this assay, all hFlex3a2_v2 variants except those containing H34A either alone or in combination displayed complement-mediated bactericidal activity against serotype 3b, reaching approximately 75% killing at 100 nM, the highest concentration of antibody tested (Fig. 7, *C* and *D*). The single variants S30G and L33N, as well as the combinatorial variants were substantially more potent in this assay than the parent mAb (Fig. 7, *C* and *D*, and Table 1). Similarly, in an OPA assay, hFlex3a2_v2 was able to facilitate opsonophagocytosis at a concentration of 30 nM, leading to 7.4% CTV + monocytes (Fig. 7*E*). At the same concentration of mAb, the most potent single amino acid variants L33N, A51R, and N90H were ∼3-fold more potent at OPA, while the most potent combinatorial variants were ∼4.5-fold more potent (Fig. 7*E*). However, despite improving affinity and potency in effector function assays, the higher affinity variants of hFlex3a2_v2 did not block *S. flexneri* 3b invasion (Fig. 7*F*). Together, this data shows that panning against *S. flexneri* 3a was able to increase the functional breadth of this antibody to increase effector functions against *S. flexneri* 3b, likely by improving the affinity to a shared epitope on these two serotypes that was weakly recognized by the parent mAb.

## Discussion

Here, we report the development of a panning strategy that enabled the rapid affinity maturation of a mAb against the O-Ag of *S. flexneri* 3a. We demonstrate that variants with improved affinity exhibit more potent effector functions against both *S. flexneri* 3a and the related but distinct serotype *S. flexneri* 3b. By pairing whole-cell in-solution panning with NGS and using both the target bacterial strain and a related strain with a completely unrelated O-Ag, we were able to efficiently identify specific mutations that could increase affinity against the target strain. It is notable that out of the four tested variants that met the criteria for specific enrichment (S30G, L33N, A51R, and N90H), three of them (S30G, L33N, A51R) improved affinity by ELISA, and all four exhibited improved potency in at least one effector function assay. One of these mutations, L33N, was sufficient to increase apparent affinity in ELISA by approximately 10-fold over the parent mAb, which is comparable to the relative differences in affinity of single amino acid variants that have been reported for germline mAbs against *Chlamydia* core lipopolysaccharide (29). In contrast, H34A, which was enriched against both bacterial species and is predicted to be located at the VH/VL interface (Fig. 5*E*) (possibly indicating that this mutation improves a non-specific parameter such as display efficiency on phage), had lower affinity than the parent, supporting the rationale of this approach.

Unexpectedly, while variants in the hFlex3a2 VL library were successfully enriched following panning, no variants were enriched from the VH library (Fig. S1). It is possible that the VH is already optimized, and we were simply unable to find point mutations that conferred additional advantage. This is supported by the observation that, while the VL protein sequence of hFlex3a2 is identical to germline, the VH sequence already contains somatic mutations that result in four amino acid substitutions (Fig. 1*A*). However, the fact that no enrichment was observed against either *S. flexneri* 3a or *S. sonnei* (which would not be expected, as at least some variants should be nonspecifically enriched for reasons of improved display efficiency or scFv stability) leaves open the possibility that this was a technical issue with the panning process rather than a biological one. Further experiments would be needed to definitively determine whether any additional VH mutations can further improve the binding and function of hFlex3a2. The identification and characterization of VH mutations that further synergize with the already identified VL mutations will be of interest in future studies.

Altogether, these data support the hypothesis that higher affinity generally improves antibacterial function performance for anti-O-Ag mAbs. Higher affinity hFlex3a2 variants were generally more potent across all assays (Figs. 5 and 7), with some notable exceptions. In L-ABA, affinity was correlated with potency (Table 1), suggesting that potency in this assay has a relatively linear relationship with affinity. Similarly, higher affinity variants were able to block invasion by *S. flexneri* 3a, while the parent mAb was not (Fig. 5*D*). Since the parent mAb was already potent in effector function assays against *S. flexneri* 3a, the improvements in higher affinity variants were subtle. Against *S. flexneri* 3b, where the parent mAb was only weakly functional in the L-ABA and OPA assays, more substantial improvements were observed. However, even these enhanced variants were unable to block invasion by serotype 3b (Fig. 7*F*). While it is possible that the mechanism of action by which hFlex3a2 and its variants inhibit *S. flexneri* 3a invasion does not apply to *S. flexneri* 3b, we suspect that this observation is more likely due to invasion inhibition requiring a greater affinity than L-ABA or OPA activity. Even though some hFlex3a2_v2 variants are more potent against *S. flexneri* 3b than the parental mAb, the best variants have weaker affinity and potency against serotype 3b than 3a. Given the inherent biological variability of the invasion assay compared to L-ABA or OPA, and that the parent mAb does not measurably inhibit invasion against even 3a, we think that the best variants simply lack sufficient affinity to reach the threshold where a decrease in invasion by 3b is consistently measurable in this assay. It is possible that panning against 3b with the hFlex3a2_v2 library would identify variants that further improve activity against this serotype. The relationship between OPA and affinity was more complex than those between affinity and the L-ABA. While the highest affinity variants tested here (L33N and S30G + L33N) did exhibit higher degrees of opsonophagocytosis than the parent mAb, N90H, which does not have detectably higher affinity and was not more potent in L-ABA, also improved macrophage uptake of both *S. flexneri* 3a and 3b (Figs.5*C* and 7*E*). Similarly, H34A, which has lower affinity and L-ABA potency than the parent, performed similarly to the parent mAb in the OPA (Figs.5*C* and 7*E*).

Although they are difficult to explain, the discrepancies observed between apparent affinity and effector function potency between N90H, H34A, and the others are not entirely unprecedented. A similar phenomenon has been observed for some mAbs targeting the human immunodeficiency virus (HIV) glycoprotein gp120 as they exhibit different capacities to drive antibody-dependent cellular cytotoxicity against HIV-infected cells displaying gp120, despite being from the same lineage, having the same affinity, and binding the same epitope (47). In that study, it was proposed that these differences are a consequence of either subtle variations in the structural recognition of the epitope or other changes in the binding dynamics between the mAb and the antigen. Similarly, work studying anti-CD4, anti-EGFR, and anti-HER2 mAbs found that some mAbs could exhibit more potent Fc-mediated activity against cancer cell lines than others that targeted the same epitope with higher affinity, because a faster off-rate resulted in monovalent binding and a greater density of Fc on the surface (48). We suspect similar factors, such as differences in binding kinetics, valency, or geometry may explain why N90H or H34A perform better in the OPA than would be expected based on their apparent affinities. It is also important to recognize the degree that extreme avidity is likely shaping the mAb-O-Ag interaction in all binding and functional assays presented here. Because each bacterial cell is covered in millions of LPS molecules (49), and each LPS molecule has 10 to 100 O-Ag units (50), the number of antigen binding sites available to mAbs bound to a bacterial cell are significant. Even with purified O-Ag, we see little detectable dissociation by BLI (Fig. 4*G*), suggesting that avidity effects may limit dissociation following mAb binding. This extensive avidity likely impacts the binding dynamics and orientation of anti-OAg mAbs on the bacterial surface, which in turn will shape the Fc-exposure and clustering that drive complement deposition and opsonophagocytosis.

Most importantly, this work informs the development of therapeutic mAbs targeting bacterial polysaccharides. More test cases are required to establish the generalizability of this approach. However, these data demonstrate that in principle, this whole-cell in-solution panning approach can rapidly identify variants with improved affinity without the need for antigen purification, and these improvements lead to direct functional improvements. More interestingly, the observed enhancement of activity against *S. flexneri* 3b suggests that if a mAb is targeting an epitope shared with other bacterial serotypes, it may be possible to improve functional breadth alongside potency. This supports the development of clinically relevant mAbs against bacterial polysaccharides, because discovery campaigns can now prioritize mAbs that have relatively high breadth by binding sugars common to many bacterial polysaccharides. Broad binders can then be rapidly improved using this method. Future work will explore the potential of more complex panning strategies that incorporate multiple serotypes, which can further facilitate the identification of variants with improved breadth. Interestingly, recent work suggests that the functional breadth of mAbs against bacterial polysaccharides can also be improved by expressing mAbs as IgM, because the multimeric structure of this isotype facilitates greater avidity against weakly recognized epitopes conserved across species (51). Combining this observation with the approach piloted here could enable the development of antibacterial mAbs targeting specific classes of polysaccharides conserved across species. Cocktails of affinity-matured IgM could conceivably provide potent protection across common nosocomial pathogens. Altogether, these results are an important step towards expanding the mAb engineering toolkit for anti-polysaccharide mAbs, which in turn may help develop novel therapeutics against AMR bacterial pathogens.

## Experimental procedures

### Bacterial strains and growth conditions

Bacterial strains and plasmids used in this study can be found in Table S2. *Shigella* strains were maintained at −80 °C in tryptic soy broth (TSB) containing 20% (vol/vol) glycerol. *Shigella* was grown aerobically at 37 °C on TSB agar with 0.01% Congo red dye.

For all assays, bacteria were grown in the desired medium overnight, then subcultured 1:100 into fresh growth medium and grown aerobically at 250 rpm, 37 °C.

### Cell culture conditions

HeLa cells (ATCC CCL-2.1) were grown in high glucose DMEM (Gibco) + 10% FBS + 2 mM glutamine and 1x Pen/Strep. THP-1 cells (ATCC TIB-202) were grown in RPMI (Gibco) + 10% FBS + 10 mM Hepes pH 7.4 + 1 mM Sodium Pyruvate + 55 μM beta-mercaptoethanol and 1x Pen/Strep. For assays involving bacteria, HeLa and THP-1 cells were first washed into fresh media that does not contain Pen/Strep. Expi293 cells were grown in Expi 293 Expression Medium (Gibco). THP-1 cell lines were obtained from ATCC. Stocks were frozen within five passages, and further cell line validation was not performed. *Mycoplasma* testing is performed in our laboratory on occasion but was not performed specifically for any of the cell lines used in experiments described in this paper.

### 10x sequencing of hybridomas

An unsequenced hybridoma expressing the mAb hFlex3a2, which was discovered and described previously by WRAIR and collaborators (38), was provided to IAVI by WRAIR. Single cells were isolated using a BD FACSMelody cell sorter, and heavy and light chain variable domains were sequenced using the 10X single cell genomics platform (10X Genomics, Inc.). Sequence analysis to predict CDRs, align to germline, and identify rare residues was done using IMGT/V-Quest, IgBLAST, and abYsis (52, 53, 54). The VH and VL sequences were cloned into a modified pcDNA3.2 expression vector and purified as full IgG as described below. The mAb was confirmed to have properties consistent with the parental hybridoma mAb (Fig. 1).

### Construction of plasmids

Primers and plasmids used in this study can be found in Table S3. For periplasmic expression of scFvs, hFlex3a2 and hFlex3a2_v2 were designed as scFvs in the following orientation—PelB signal sequence, hFlex3a2 VH, (GGGGS)3X linker, hFlex3a2 VL —ordered as gBlocks (Integrated DNA Technologies), then cloned between the NcoI and BamHI restriction sites of the bacterial expression vector pET28b (+) using NEBuilder HiFi DNA Assembly following the manufacturer’s instructions (New England Biolabs). For expression as huIgG1, the variable regions of hFlex3a2 and hFlex3a2_v2 were cloned into a pcDNA3.2-based human antibody expression vector containing the relevant huIgG1 or Ig kappa constant domains. For single amino acid mAb variants, primers were designed to insert the mutations into the appropriate VL sequence by HiFi DNA Assembly (New England BioLabs). Combinatorial variants of hFlex3a2 and hFlex3a2_v2 VL chains were ordered as gBlocks and inserted into the appropriate human antibody expression vector using NEBuilder HiFi assembly.

### Construction of *Shigella* mutants

*S. flexneri* 3a and 3b *tolR* mutants that hyperproduce OMVs (55), and *S. flexneri* 3a *waaL* mutant (O-Ag knockout) (56) were produced by the gene-gorging method as previously described (57). Briefly, a mutation cassette consisting of 500 bp of *Shigella* genomic DNA upstream of the target mutation site, the kanamycin resistance gene from plasmid pKD4, and 500 bp of DNA downstream of the target mutation site, flanked on both sides of the mutation cassette by the restriction site I-SceI, was constructed using splice by overlap extension PCR (58). The mutation cassette was A-tailed and ligated into the plasmid pGEM-T Easy (Promega) to create a donor plasmid. The donor plasmid was electroporated into the desired *S. flexneri* strain that had previously been electroporated with plasmid pACBSR. Strains containing both the donor plasmid and pACBSR were grown for 6 h at 30 °C in LB + chloramphenicol + 0.4% arabinose to induce genomic recombination and then plated on TSBA + Kanamycin for single colonies. Colonies were passaged multiple times at 37 °C to promote the loss of pACBSR. After multiple passages, colonies were confirmed to be kanamycin resistant but chloramphenicol sensitive, indicating loss of the donor plasmid, and the successful insertion of the genomic mutation was verified by Sanger sequencing.

### Phage library construction and panning

Double-barcoded site-saturated mutagenesis scanning libraries of the VL and VH of hFlex3a2_v2 were designed and ordered from Twist Biosciences. The libraries were amplified with primers to introduce SfiI sites and then ligated into the SfiI site of phagemid pCOMB3XSS (59). To produce phage, the phagemid libraries were electroporated into XL1-Blue (Agilent), recovered in 5 ml of SOC, and then grown (37 °C, 250 RPM) to OD_600_ ∼0.5 in 10 ml of Super Broth + Tet + Carb before being super infected with M13ko7 helper phage (New England Biosciences) at an MOI of 20, expanded, and grown overnight (30 °C, 250 rpm) in 200 ml of super broth + Tet + Carb + Kan.

Phage was precipitated from the supernatant with 0.5 M NaCl and 4% PEG-8000 as previously described (60) and then panned against whole bacteria in-solution, based on a previously published method (44). Briefly, 1 × 10^9^ cfu of *S. flexneri* 3a or *S. sonnei* were blocked in 3% nonfat milk in PBS and then each bacterial species was incubated with 5 × 10^10^ pfu of blocked phage particles for 15 min to 2 h. Non-binding phage was washed 5x with PBS + 5% Tween-20 after round 1 of panning and washed 10x after round 2. The binding phage was eluted by mixing with 0.1 M HCl pH 2.2 (pH adjusted with glycine) for 10 minutes at room temperature, then neutralized with 2 M Tris base and used to reinfect XL1-Blue cells to generate phage for the second round of panning. XL1-Blue containing the input library, post-panning round 1 phagemids, and post-panning round two phagemids were maxiprepped, the variable chain sequences were PCR-amplified and deep-sequenced by MiSeq (Illumina) using Amplicon-EZ platform (Genewiz). The relative enrichment of phage library variants against *S. flexneri* 3A and *S. sonnei* after rounds 1 and two of panning was quantified using an in-house Python script (43). The scripts can be found at https://github.com/jardinelab/Multistate-optimization-of-HIV-bnAbs.

### Antibody and antigen purification

For purification of IgGs, Expi293 cells were co-transfected with the desired heavy chain and light chain pair at a ratio of 1:2.5 with FectoPRO (Polyplus) in Expi293 Expression Medium (Gibco). The cells were supplemented with 3 mM valproic acid (Sigma-Aldrich) and 0.4% D-(+)- glucose (Sigma-Aldrich) 24 h after transfection. The supernatant was harvested on day 5 post-transfection, IgGs were purified using Praesto AP + agarose resin (Purolite), and then buffer exchanged and concentrated using a 30 kDa cutoff spin filter (Amicon).

scFv purification was based on a previously described method (61). Briefly, BL21 containing the desired scFv cloned into pET28b (+) was grown to OD_600_ 0.6 to 0.8 in terrific broth (37 °C, 250 rpm) and then induced with 1 mM IPTG overnight (18 °C, 250 RPM). Bacteria were collected, resuspended in 40 ml of TES buffer (0.5 M Sucrose, 0.5 mM EDTA, and 0.2 M Tris-HCl pH 8.0), incubated at 4 °C for 30 min, and then osmotically shocked with 80 ml of ice-cold ddH_2_O for 1 h at 4 °C. Spheroplasts were pelleted, the supernatant was collected, adjusted to 150 mM NaCl, 2 mM MgCl_2_, 20 mM imidizaole, and incubated with 1 ml of Ni-NTA resin (Anatrace) at 4 °C for at least 1.5 h scFv bound resin was packed onto gravity columns, washed extensively with wash buffer (150 mM NaCl, 2 mM MgCl_2_, 30 mM imidazole), and then eluted with elution buffer (150 mM NaCl, 2 mM MgCl_2_, 300 mM imidazole) before buffer exchanging and concentrating with PBS using a 10 kDa cutoff centrifugal filter (Amicon).

To purify OMVs, supernatant was collected from *S. flexneri* 3a or 3b tolR::kan mutants grown overnight in SSDM (55) supplemented with 40 mg/L l-methionine and 20 mg/L tryptophan. The supernatant was filter-sterilized through a 0.2 μM filter (Sartorius) and then concentrated to ∼ 50 ml by tangential flow filtration using a Vivaflow 100 kDa cutoff filter (Sartorius). The concentrated supernatant was ultracentrifuged at 200,000*g* for 1.5 h to pellet OMVs, which were then resuspended in 1 ml of PBS and stored at −20 °C for future use. Total protein content of OMVs was quantified by Micro-BCA assay (Thermo Fisher) following the manufacturer’s instructions. OMV amount was normalized by total protein content for ELISA assays.

O-Ag was purified from whole bacteria by acetic acid hydrolysis (62). Acetic acid was added at a final concentration of 2% to a culture of stationary phase *S. flexneri* 3a and incubated at 100 °C for 5 h. After hydrolysis, the culture was neutralized to pH ∼ 6 with ammonium hydroxide (Sigma-Aldrich). Bacterial debris was pelleted, the supernatant was buffer exchanged by tangential flow filtration using a VivaFlow 30 kDa cutoff filter (Sartorius) first into 1 M NaCl, and then into ddH_2_O.The buffer exchanged supernatant was concentrated to ∼50 ml. Citrate buffer (20 mM, pH 3) was added to the supernatant, and it was incubated at room temperature for 30 min, centrifuged at 12,000*g* for 30 min and then passed through a Sartobind IEX S75 cationic exchange filter (Sartorius). After cation exchange, the supernatant was precipitated once more with 18 mM Na_2_HPO_4_, 24% ethanol, and 200 mM CaCl_2_ at room temperature for 30 min, then buffer exchanged as described above before being concentrated to approximately 1 ml in ddH_2_O using a 30 kDa cutoff spin filter (Amicon). The purified O-Ag was quantified using a Total Carbohydrate Assay Kit (Sigma-Aldrich) following the manufacturer’s instructions. Contaminating DNA and protein levels were assessed by nanodrop analysis and Micro-BCA assay, respectively, and confirmed to be at least 10-fold lower than carbohydrate concentration.

### ELISA assays

All ELISA assays were performed in 384-well high-binding ELISA plates (Corning). Wells were coated overnight at 4 °C with antigen in PBS at a concentration of 2.5 μg/ml for OMVs or 5 μg/ml for IpaD, ovalbumin, CHO-SMP, insulin, and ssDNA. Antigen-coated wells were blocked in PBS containing 3% BSA and 0.05% Tween-20 at 37 °C for 1 h. Antibodies to be tested were diluted in 1% BSA + 0.017% Tween-20 at the desired antibody concentration and incubated with the blocked antigen at 37 °C for 1 h. After incubation with the test antibody, wells were washed 3× with PBS + 0.05% Tween-20 and incubated with the appropriate secondary antibody at room temperature for 1 h. Secondary antibodies used were either AP-conjugated anti-Flag M2 (Sigma-Aldrich) for scFvs or AP-conjugated anti-Human Fc (Jackson ImmunoResearch) for huIgG1s, both at a 1:1000 dilution in PBS + 1% BSA + 0.017% Tween-20. After washing three times more, AP substrate tablets (Sigma-Adrich) were dissolved and diluted to 1 mg/ml in AP buffer (50 mM MgCl_2_, 100 mM NaCl, 1%Tween 20). ELISAs were developed with AP substrate (20 min for IgGs or 40 min for scFvs) at room temperature. Absorbance at 405 nm was read on a BioTek Synergy microplate reader. mAb D.02-E4 formatted as a human IgG1 was used as a control mAb (25).

### Luminescent antibody bactericidal assays (L-ABA)

L-ABA was adapted from previously published protocols (38, 63). Briefly, the desired *S. flexneri* serotype was grown to mid-log phase in TSB (BD Biosciences) and resuspended at a concentration of 3 × 10^5^ CFU/ml in 100 μl of assay buffer (HBSS + 0.1% Gelatin, pH 7.4) with 7.5% Baby Rabbit Complement (Pel-Freez Biologicals), and the test antibody at the desired concentration. The L-ABA reaction was incubated for 3 h at 37 °C, washed once with 100 μl of fresh assay buffer, mixed 1:1 with BacTiter-Glo (Promega), and incubated for 5 min at room temperature. Luminescence was read on a BioTek Synergy plate reader. Adalimumab (64) expressed as a chimeric macaque IgG1 or mAb1416, and human IgG1 (8) were used as control mAbs in L-ABAs against *S. flexneri* 3a and 3b, respectively. To normalize for differences in bacterial growth, the data were fit with a sigmoidal four-parameter logistic curve on GraphPad Prism, and each curve was normalized to the Top best-fit value of their curve to determine their fraction maximal luminescence. Control mAb values are normalized to the luminescence signal of total bacterial growth in assay buffer and baby rabbit complement alone.

### Opsonophagocytosis assays (OPA)

For OPA, the desired *S. flexneri* strain was grown to OD_600_ of approximately 0.5 in TSB, washed with PBS, and stained with a 1:50 dilution of CellTrace Violet (CTV, Thermo Fisher) for 15 min at 37 °C. Excess dye was quenched with FACS buffer (PBS + 1% FBS), the bacteria were washed in PBS and resuspended in assay buffer (HBSS + 0.1% gelatin, pH 7.4). 2.5 × 10^6^ CFU of bacteria were mixed with the desired concentration of mAb in assay buffer and incubated at 37 °C for 30 min. Opsonized bacteria were then mixed with 5 × 10^4^ undifferentiated THP-1 cells and incubated for 30 min at 37 °C in 5% CO_2_. After 30 min, the OPA reactions were stained on ice in the dark for 30 min with Live/Dead Near-IR dye (Thermo Fisher) diluted 1:2000 in PBS and FITC-conjugated anti-CD45 antibody (BD Biosciences) diluted 1:4000. The cells were then fixed with 4% PFA for 30 min, on ice. To determine relative OPA activity, the percentage of THP-1 cells incubated with *S. flexneri* opsonized with the mAbs of interest that were Near IR-, CD45+, and CTV+ was quantified by flow cytometry using a FACSCelesta (BD Biosciences). Adalimumab (64) expressed as a chimeric macaque IgG1 was used as a control mAb.

### Invasion assays

Invasion was measured by a gentamicin protection assay (65). *S. flexneri* was grown to an OD_600_ of approximately 0.7 in TSB (37 °C, 250 RPM), washed in PBS, resuspended at a concentration of 1 × 10^9^ CFU/ml, and incubated with 1 μM of the desired mAb or PBS (control) for 30 min at 37 °C. 10 μl of bacteria (∼1 × 10^7^ CFU, MOI = 20) was added to a monolayer of approximately 5 × 10^5^ HeLa cells in a tissue culture-treated 24-well plate (Corning) and centrifuged at 900*g* for 10 min. The plate was incubated for 30 min at 37 °C and 5% CO_2_, washed two times with PBS, the media was replaced with DMEM containing 25 μg/ml gentamicin, and the plate was incubated for an additional 1 h. The wells were washed twice more with PBS and then lysed with 0.1% Triton X-100. Serial dilutions of the mAb-treated and control bacteria were plated, and the percentage of invasion was calculated. The invasion rate of mAb-treated *S. flexneri* 3a was normalized to the invasion rate of *S. flexneri* 3a in PBS alone. Adalimumab (64) expressed as a chimeric macaque IgG1, was used as a control mAb.

### Bacterial surface staining

To screen for bacterial surface staining, a panel of *Shigella* strains was grown to an OD_600_ of approximately 0.5, washed and resuspended in PBS to a concentration of 2 × 10^9^ CFU/ml. The desired mAb was added to 25 μl of bacteria at a final concentration of 50 nM and incubated at 37 °C for 30 min. The bacteria were washed with PBS and then incubated with a 1:100 dilution of the appropriate secondary antibody for 30 min at room temperature. For scFvs, the secondary antibody was Alexa Fluor 647-conjugated anti-Flag antibody (Abcam), and for huIgGs this was BV421-conjugated anti-Human IgG (BD Biosciences). Bacteria were then fixed with 4% PFA for 30 min on ice. Median fluorescence intensity was determined by flow cytometry on a FACSSymphony A5 (BD Biosciences).

To compare relative surface staining of hFlex3a2_v2 variants, *S. flexneri* 3a or 3b were grown as described above, then resuspended in PBS to a concentration of 2 × 10^7^ CFU/ml. The desired mAb was added to 100 μl of bacteria at the indicated concentrations in a total volume of 200 μl and incubated at 37 °C for 1 h. The bacteria were washed with PBS and then incubated with a 1:100 dilution of BV421-conjugated anti-Human IgG (BD Biosciences) for 30 min at room temperature. Bacteria were then fixed with 4% PFA for 30 min on ice and the percent of bacteria positive for antibody binding was determined by flow cytometry on a FACSSymphony A5 (BD Biosciences). mAb1416 expressed as a human IgG1 (8) was used as a control mAb.

### Biolayer interferometry

Binding of hFLex3a2_v2 variants to *S. flexneri* 3a O-Ag was characterized by BLI using an Octet R8 (Sartorius) with Octet AHC Biosensors (Sartorius). All antibodies and antigens were in PBS + 0.02% Tween-20 + 0.01% BSA. The BLI protocol was as follows: 300 s baseline, 120 s loading with 1 μM mAb, 180 s baseline, 360 s association with 100 μg/ml of *S. flexneri* 3a O-Ag or 540 s with 20 μg/ml of *S. flexnei* 3a O-Ag, and 600 s dissociation. BLI results were analyzed on Octet Analysis Studio (Sartorius) and plotted with GraphPad Prism. hFlex2a1 (38) expressed as a chimeric huIgG1 was used as a control mAb.

### O-Ag visualization

To visualize O-Ag, crude protein-free LPS extract was purified from *S. flexneri* 3a, *S. flexneri* 3a waaL::kan (O-Ag-), or *S. flexneri* 3a waaL::kan pwaaL induced with 1 mM IPTG following the method of Darveau and Hancock (66). Bacterial LPS extract was separated by SDS-PAGE and stained with Pro-Q Emerald 300 Lipopolysaccharide Gel Stain Kit (Thermo Fisher) following the manufacturer’s instructions. Stained gels were visualized on a ChemiDoc MP Imaging System (Bio-Rad).

### Protein structure prediction

To predict a structural model of hFlex3a2 VH and VL, we use Alphafold2 through ColabFold (46). The VL and VH sequences for wildtype hFlex3a2 were input into ColabFold in a Google Colab environment, and Alphafold multimer v3 was used to predict the complex. The five output structures differed from one another by less than 0.2 Å^2^, without apparent differences in the CDR structures. Figures were made using PyMol.

## Data availability

All relevant data have been included in this manuscript. Raw data for images or phage library sequencing is available upon request.

## Supporting information

This article contains supporting information (57, 59, 67, 68).

## Conflict of interest

The authors declare that they do not have any conflicts of interest with the content of this article.
