# Supplementary material for: Improvement in binding and function of a monoclonal antibody against Shigella flexneri 3a O-antigen via phage display and whole-cell in-solution panning
Source: J Biol Chem. 2026 Mar 25;302(5):111405. doi: 10.1016/j.jbc.2026.111405 (PMC13098420; doi:10.1016/j.jbc.2026.111405)
Supplement: Figure S1 [file mmc1.pptx]

## Slide 1
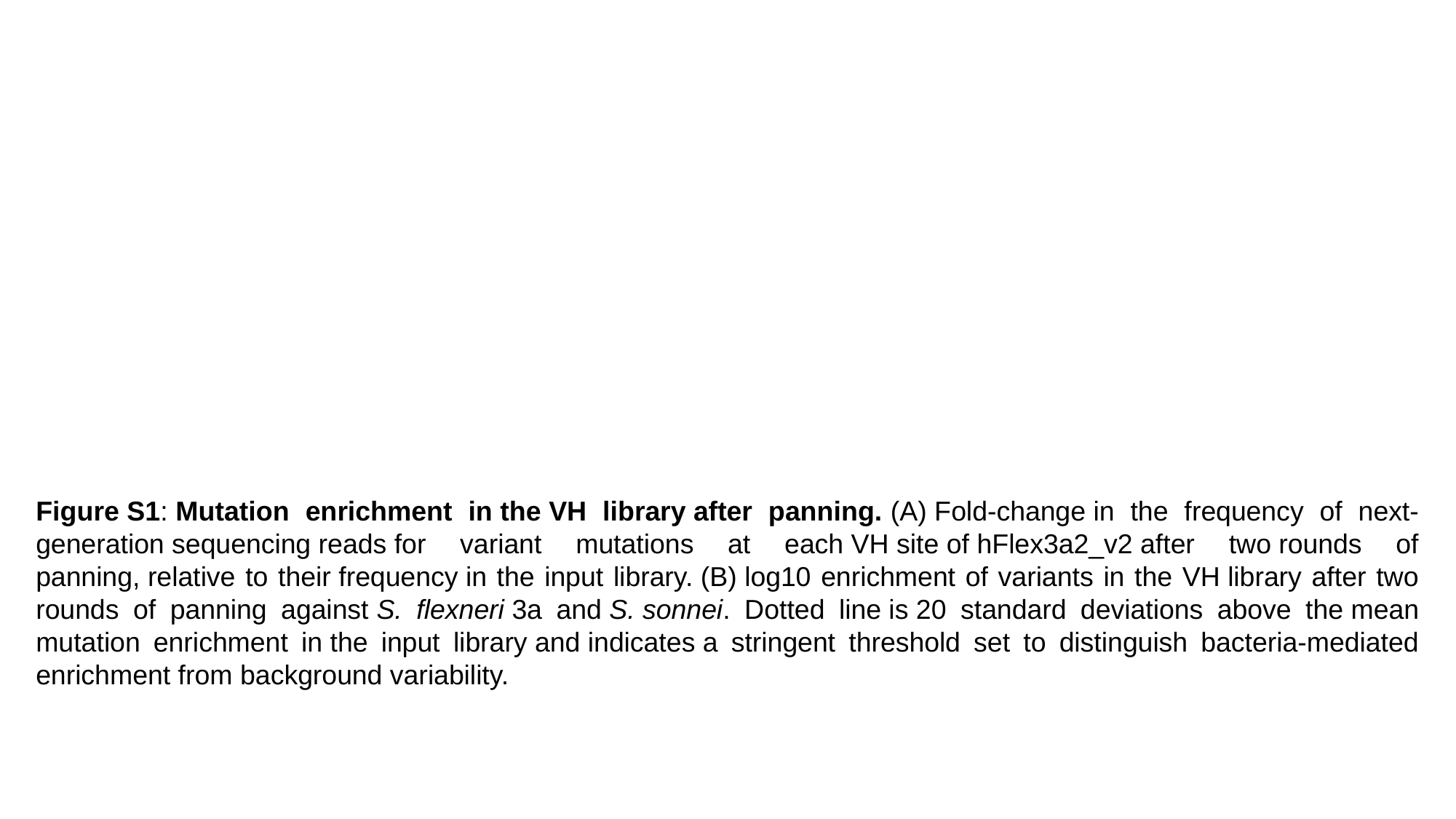

Figure S1: Mutation enrichment in the VH library after panning. (A) Fold-change in the frequency of next-generation sequencing reads for variant mutations at each VH site of hFlex3a2_v2 after two rounds of panning, relative to their frequency in the input library. (B) log10 enrichment of variants in the VH library after two rounds of panning against S. flexneri 3a and S. sonnei. Dotted line is 20 standard deviations above the mean mutation enrichment in the input library and indicates a stringent threshold set to distinguish bacteria-mediated enrichment from background variability.
