# Supplementary material for: Improvement in binding and function of a monoclonal antibody against Shigella flexneri 3a O-antigen via phage display and whole-cell in-solution panning
Source: J Biol Chem. 2026 Mar 25;302(5):111405. doi: 10.1016/j.jbc.2026.111405 (PMC13098420; doi:10.1016/j.jbc.2026.111405)
Supplement: Figure S2 [file mmc2.pptx]

## Slide 1
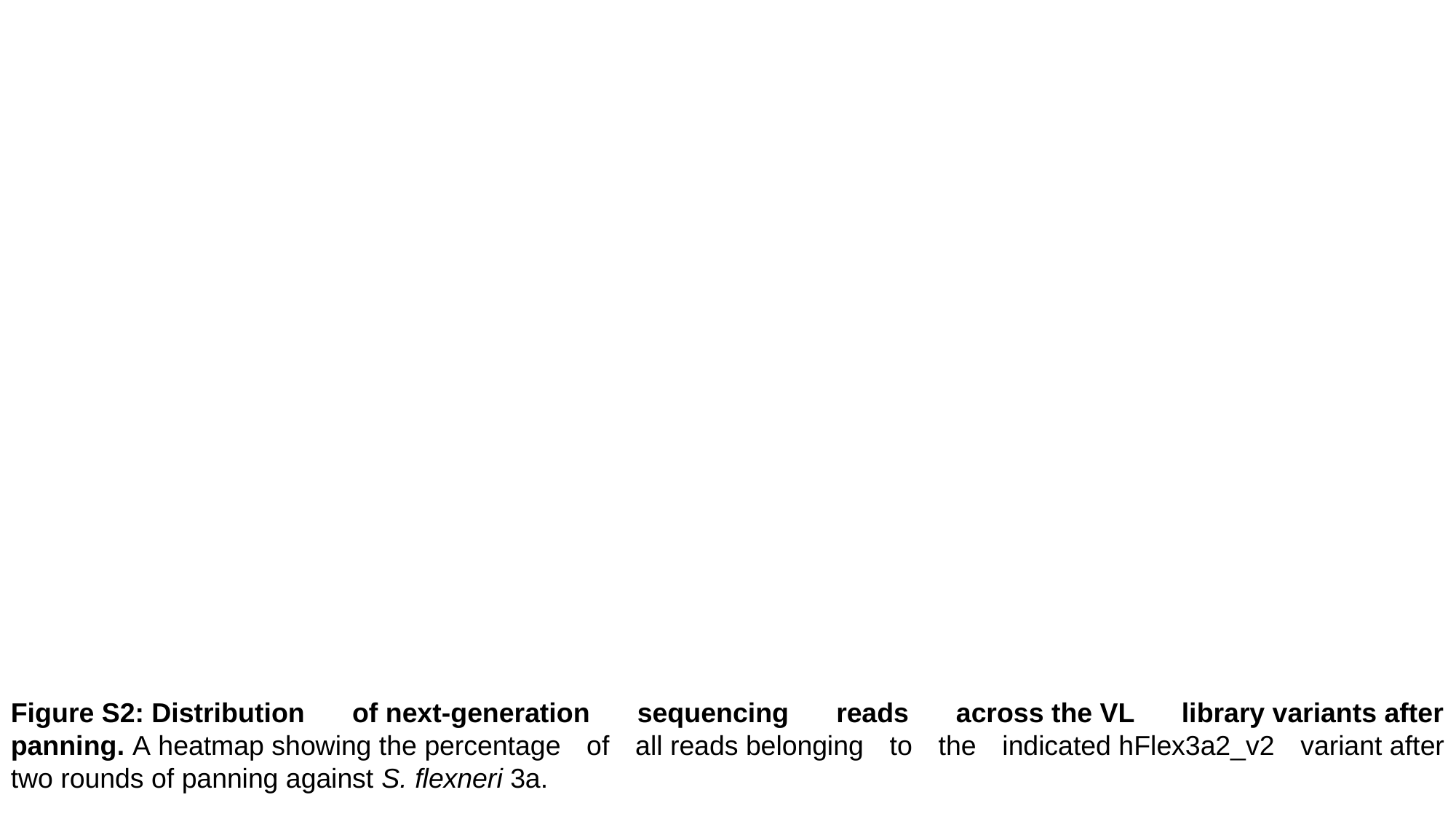

Figure S2: Distribution of next-generation sequencing reads across the VL library variants after panning. A heatmap showing the percentage of all reads belonging to the indicated hFlex3a2_v2 variant after two rounds of panning against S. flexneri 3a.
