# Supplementary material for: Improvement in binding and function of a monoclonal antibody against Shigella flexneri 3a O-antigen via phage display and whole-cell in-solution panning
Source: J Biol Chem. 2026 Mar 25;302(5):111405. doi: 10.1016/j.jbc.2026.111405 (PMC13098420; doi:10.1016/j.jbc.2026.111405)
Supplement: Figure S3 [file mmc3.pptx]

## Slide 1
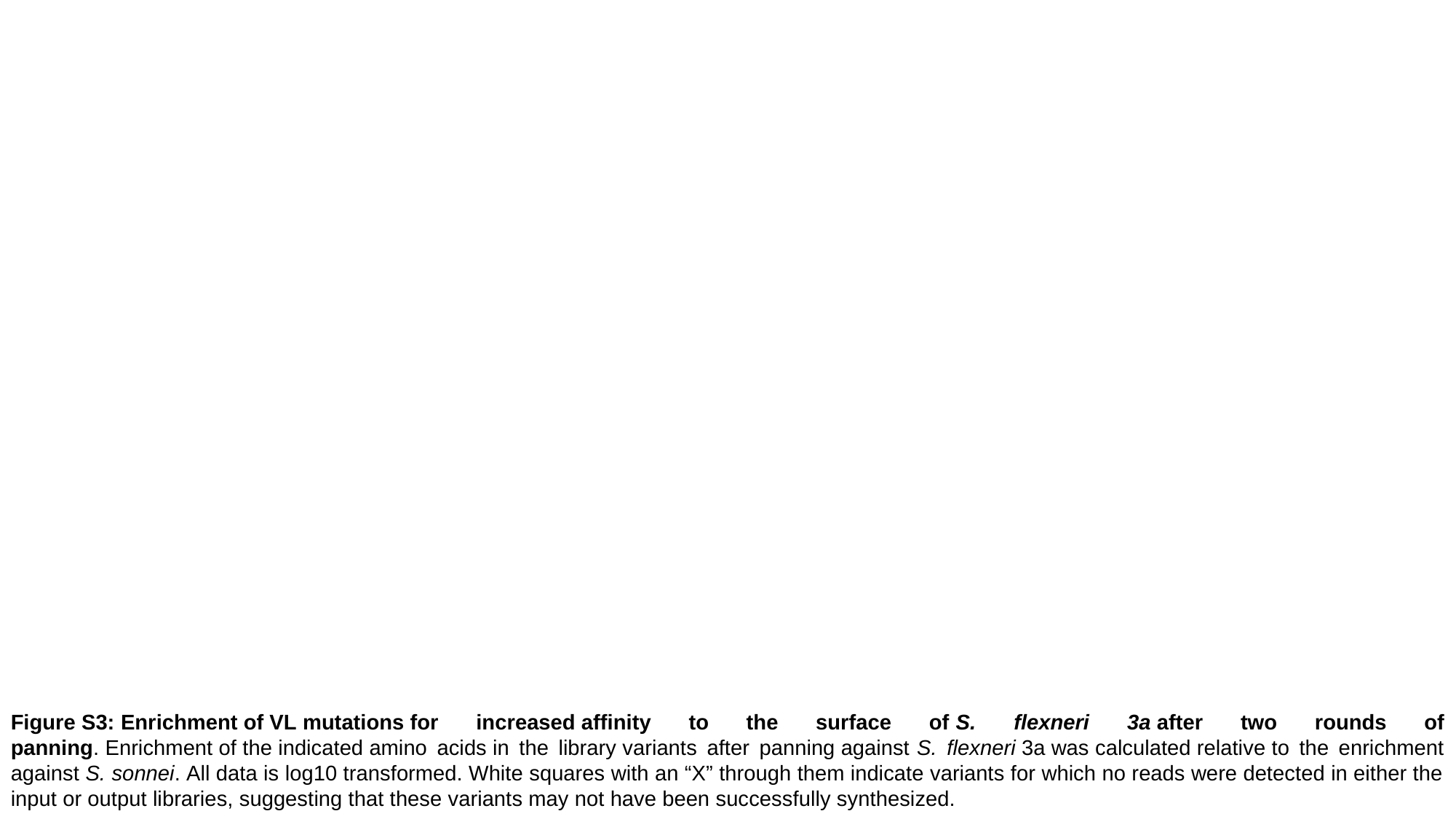

Figure S3: Enrichment of VL mutations for increased affinity to the surface of S. flexneri 3a after two rounds of panning. Enrichment of the indicated amino acids in the library variants after panning against S. flexneri 3a was calculated relative to the enrichment against S. sonnei. All data is log10 transformed. White squares with an “X” through them indicate variants for which no reads were detected in either the input or output libraries, suggesting that these variants may not have been successfully synthesized.
