# Supplementary material for: Improvement in binding and function of a monoclonal antibody against Shigella flexneri 3a O-antigen via phage display and whole-cell in-solution panning
Source: J Biol Chem. 2026 Mar 25;302(5):111405. doi: 10.1016/j.jbc.2026.111405 (PMC13098420; doi:10.1016/j.jbc.2026.111405)
Supplement: Figure S4 [file mmc4.pptx]

## Slide 1
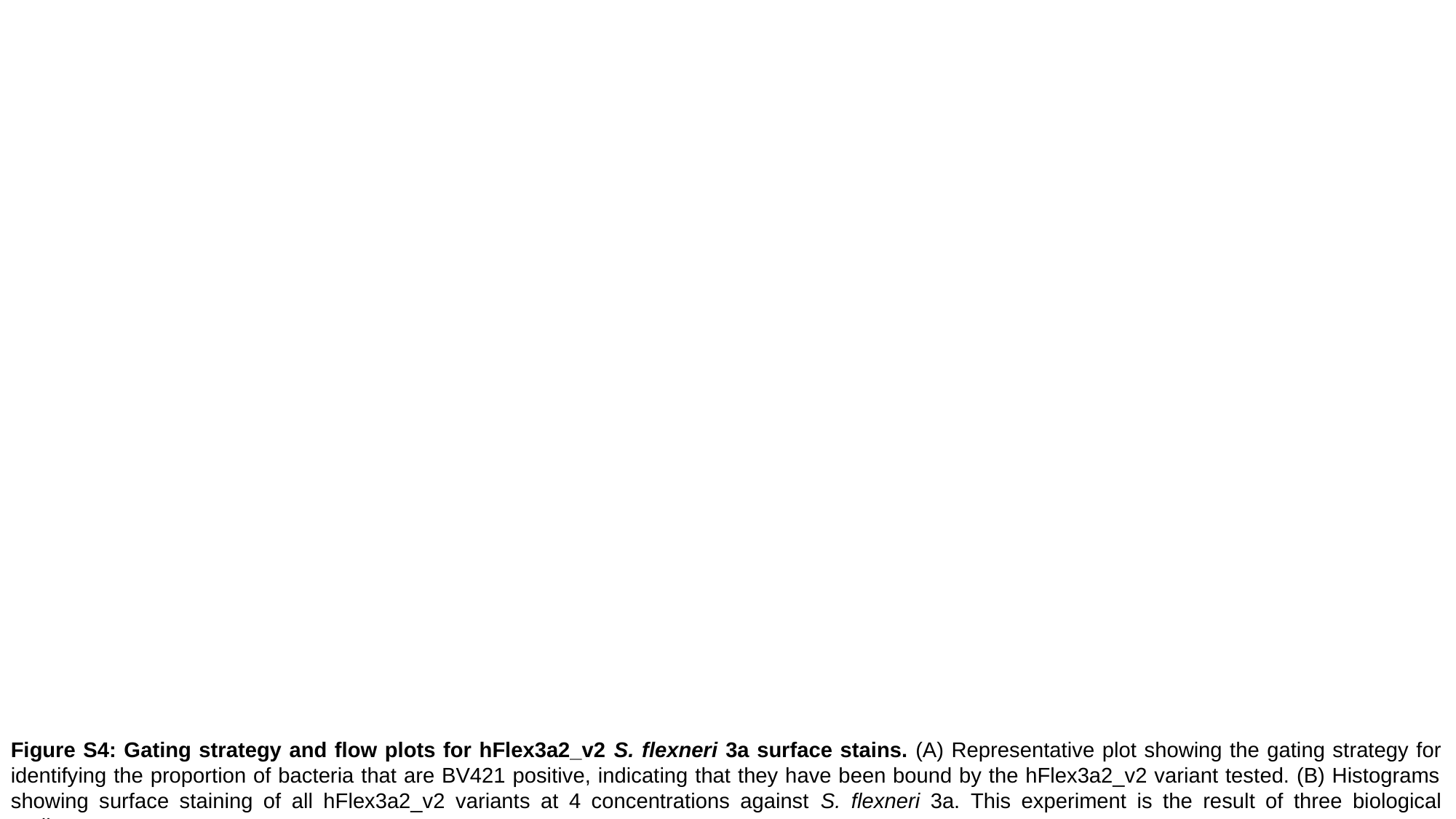

Figure S4: Gating strategy and flow plots for hFlex3a2_v2 S. flexneri 3a surface stains. (A) Representative plot showing the gating strategy for identifying the proportion of bacteria that are BV421 positive, indicating that they have been bound by the hFlex3a2_v2 variant tested. (B) Histograms showing surface staining of all hFlex3a2_v2 variants at 4 concentrations against S. flexneri 3a. This experiment is the result of three biological replicates.
