# Supplementary material for: Improvement in binding and function of a monoclonal antibody against Shigella flexneri 3a O-antigen via phage display and whole-cell in-solution panning
Source: J Biol Chem. 2026 Mar 25;302(5):111405. doi: 10.1016/j.jbc.2026.111405 (PMC13098420; doi:10.1016/j.jbc.2026.111405)
Supplement: Figure S5 [file mmc5.pptx]

## Slide 1
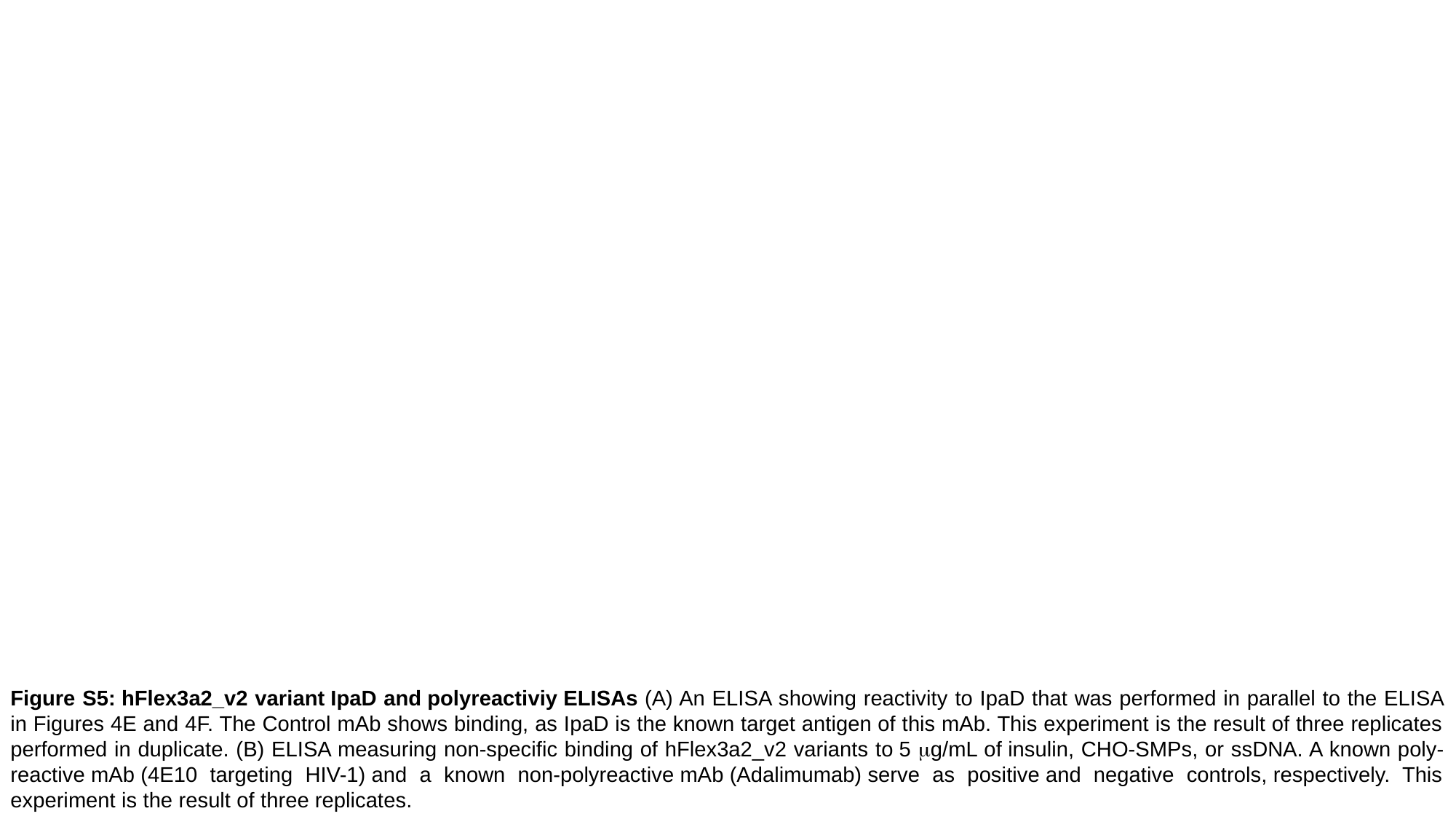

Figure S5: hFlex3a2_v2 variant IpaD and polyreactiviy ELISAs (A) An ELISA showing reactivity to IpaD that was performed in parallel to the ELISA in Figures 4E and 4F. The Control mAb shows binding, as IpaD is the known target antigen of this mAb. This experiment is the result of three replicates performed in duplicate. (B) ELISA measuring non-specific binding of hFlex3a2_v2 variants to 5 mg/mL of insulin, CHO-SMPs, or ssDNA. A known poly-reactive mAb (4E10 targeting HIV-1) and a known non-polyreactive mAb (Adalimumab) serve as positive and negative controls, respectively. This experiment is the result of three replicates.
