# Supplementary material for: Improvement in binding and function of a monoclonal antibody against Shigella flexneri 3a O-antigen via phage display and whole-cell in-solution panning
Source: J Biol Chem. 2026 Mar 25;302(5):111405. doi: 10.1016/j.jbc.2026.111405 (PMC13098420; doi:10.1016/j.jbc.2026.111405)
Supplement: Figure S6 [file mmc6.pptx]

## Slide 1
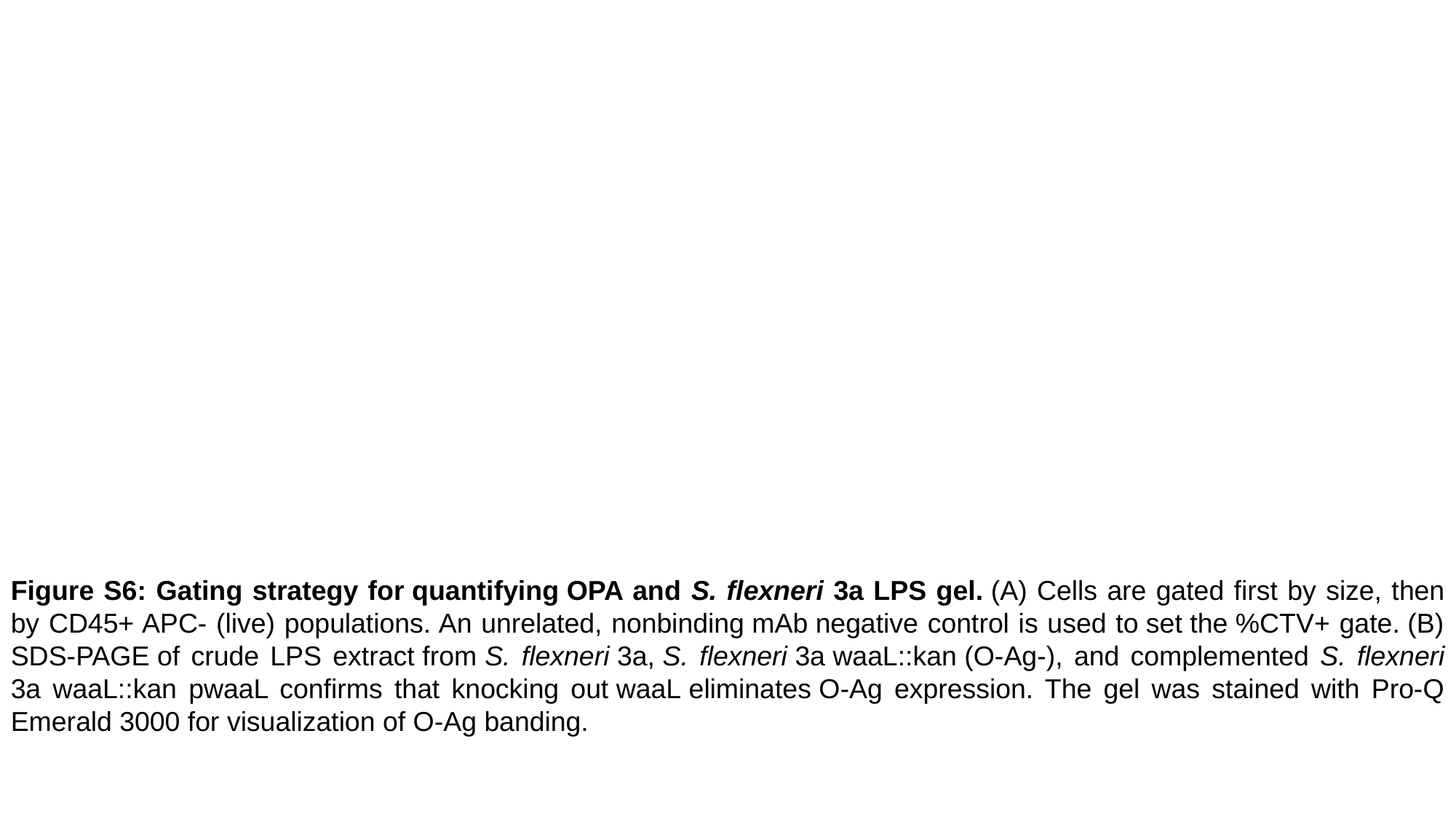

Figure S6: Gating strategy for quantifying OPA and S. flexneri 3a LPS gel. (A) Cells are gated first by size, then by CD45+ APC- (live) populations. An unrelated, nonbinding mAb negative control is used to set the %CTV+ gate. (B) SDS-PAGE of crude LPS extract from S. flexneri 3a, S. flexneri 3a waaL::kan (O-Ag-), and complemented S. flexneri 3a waaL::kan pwaaL confirms that knocking out waaL eliminates O-Ag expression. The gel was stained with Pro-Q Emerald 3000 for visualization of O-Ag banding.
