# Supplementary material for: Improvement in binding and function of a monoclonal antibody against Shigella flexneri 3a O-antigen via phage display and whole-cell in-solution panning
Source: J Biol Chem. 2026 Mar 25;302(5):111405. doi: 10.1016/j.jbc.2026.111405 (PMC13098420; doi:10.1016/j.jbc.2026.111405)
Supplement: Figure S7 [file mmc7.pptx]

## Slide 1
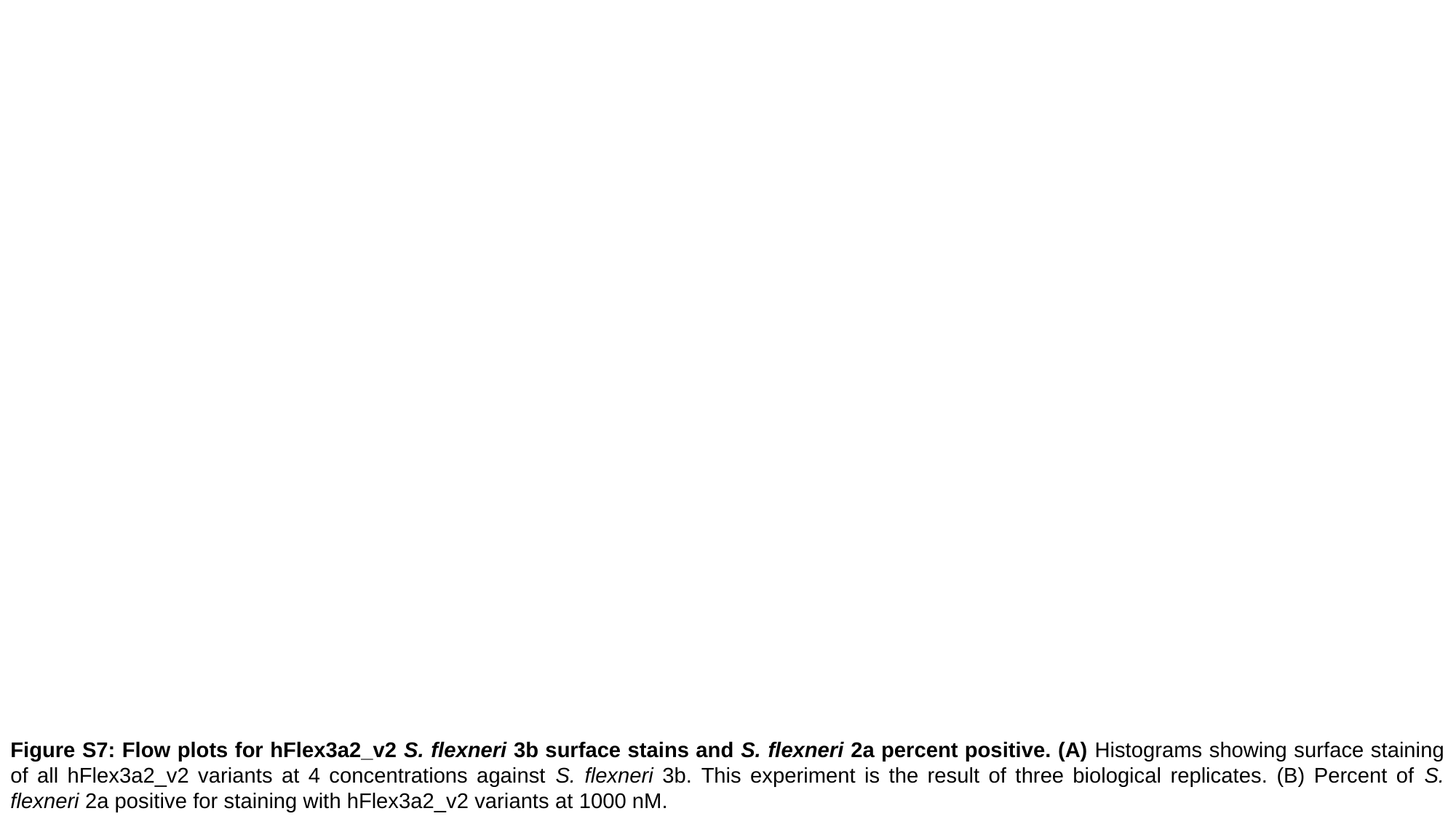

Figure S7: Flow plots for hFlex3a2_v2 S. flexneri 3b surface stains and S. flexneri 2a percent positive. (A) Histograms showing surface staining of all hFlex3a2_v2 variants at 4 concentrations against S. flexneri 3b. This experiment is the result of three biological replicates. (B) Percent of S. flexneri 2a positive for staining with hFlex3a2_v2 variants at 1000 nM.
