# Supplementary material for: Improvement in binding and function of a monoclonal antibody against Shigella flexneri 3a O-antigen via phage display and whole-cell in-solution panning
Source: J Biol Chem. 2026 Mar 25;302(5):111405. doi: 10.1016/j.jbc.2026.111405 (PMC13098420; doi:10.1016/j.jbc.2026.111405)
Supplement: Figure S8 [file mmc8.pptx]

## Slide 1
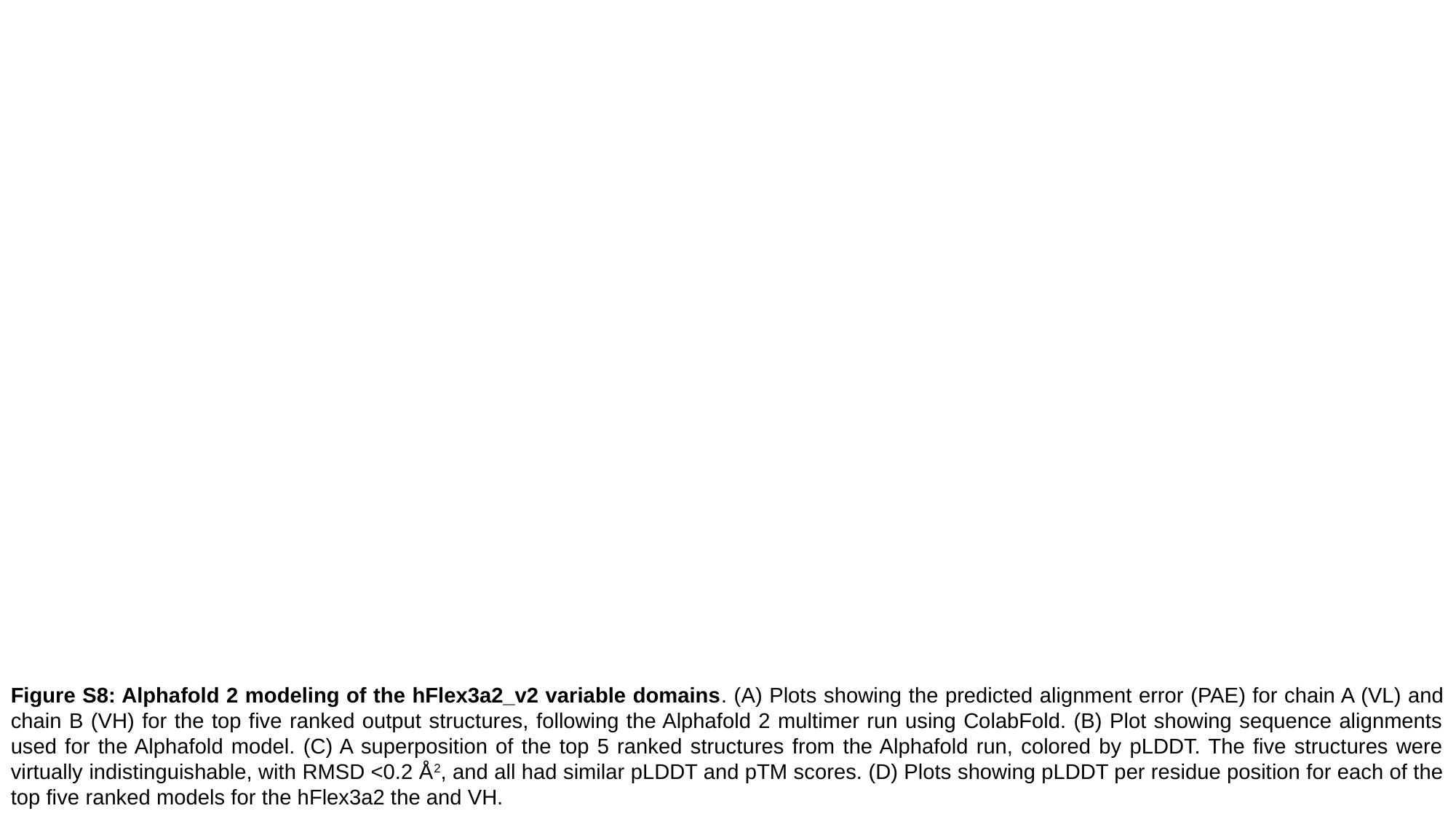

Figure S8: Alphafold 2 modeling of the hFlex3a2_v2 variable domains. (A) Plots showing the predicted alignment error (PAE) for chain A (VL) and chain B (VH) for the top five ranked output structures, following the Alphafold 2 multimer run using ColabFold. (B) Plot showing sequence alignments used for the Alphafold model. (C) A superposition of the top 5 ranked structures from the Alphafold run, colored by pLDDT. The five structures were virtually indistinguishable, with RMSD <0.2 Å2, and all had similar pLDDT and pTM scores. (D) Plots showing pLDDT per residue position for each of the top five ranked models for the hFlex3a2 the and VH.
