# Supplementary material for: Improvement in binding and function of a monoclonal antibody against Shigella flexneri 3a O-antigen via phage display and whole-cell in-solution panning
Source: J Biol Chem. 2026 Mar 25;302(5):111405. doi: 10.1016/j.jbc.2026.111405 (PMC13098420; doi:10.1016/j.jbc.2026.111405)
Supplement: Table S1 [file mmc9.docx]

**Table S1**. Amino acid sequences of hFlex3a2 and hFlex3a2_v2. Underlined residues indicate sites that are different between hFlex3a2 and hFlex3a2_v2. Red residues indicate sites of amino acid changes for experimentally tested variants identified through phage panning: S30G, L33N, H34A, A51R, and N90H.

| hFlex3a2 protein sequence | |
| --- | --- |
| Heavy Chain | QVQLQQSAPELARPGASVKMSCKASGYTFTSYTIHWVKQRPGQGLEWIGYISPSSGYTEYNQKFKDKTTLTADKSSITAYMQLSSLTSEDSAVYYCARLDNNYVYFDYWGLGTTLTVSS |
| Light Chain | DIVMTQSPATLSVTPGDRVSLSCRASQSISDYLHWYQQKSHESPRLLIKYASQSISGIPSRFSGSGSGSDFTLSINSVEPEDVGVYYCQNGHSFPLTFGAGTKLELK |
| hFlex3a2_v2 protein sequence | |
| Heavy Chain | QVQLQQS**G**PELARPGASVKMSCKASGYTFTSYTIHWVKQRPGQGLEWIGYISPSSGYTEYNQKFKDKTTLTADKSS**S**TAYMQLSSLTSEDSAVYYCARLDNNYVYFDYWGLGTTLTVSS |
| Light Chain | DIVMTQSPATLSVTPGDRVSLSCRASQSISDYLHWYQQKS**G**ESPRLLIKYASQSISGIPSRFSGSGSGSDFTLSINSVEPEDVGVYYCQNGHSFPLTFGAGTKLELK |
